# Association Between Pretransplant Dialysis Modality and Kidney Transplant Outcomes: A Systematic Review and Meta-analysis

**DOI:** 10.1001/jamanetworkopen.2022.37580

**Published:** 2022-10-20

**Authors:** Tanun Ngamvichchukorn, Chidchanok Ruengorn, Kajohnsak Noppakun, Kednapa Thavorn, Brian Hutton, Manish M. Sood, Greg A. Knoll, Surapon Nochaiwong

**Affiliations:** 1 Division of Nephrology, Department of Medicine, Faculty of Medicine Vajira Hospital, Navamindradhiraj University, Bangkok, Thailand; 2 Department of Pharmaceutical Care, Faculty of Pharmacy, Chiang Mai University, Chiang Mai, Thailand; 3 Pharmacoepidemiology and Statistics Research Center, Faculty of Pharmacy, Chiang Mai University, Chiang Mai, Thailand; 4 Division of Nephrology, Department of Internal Medicine, Faculty of Medicine, Chiang Mai University, Chiang Mai, Thailand; 5 Ottawa Hospital Research Institute, Ottawa Hospital, Ottawa, Ontario, Canada; 6 ICES uOttawa, Ottawa, Ontario, Canada; 7 School of Epidemiology and Public Health, Faculty of Medicine, University of Ottawa, Ottawa, Ontario, Canada; 8 Division of Nephrology, Department of Medicine, University of Ottawa, Ottawa, Ontario, Canada

## Abstract

**Importance:**

The benefits and disadvantages of different pretransplant dialysis modalities and their posttransplant outcomes remain unclear in contemporary kidney transplant care.

**Objective:**

To summarize the available evidence of the association of different pretransplant dialysis modalities, including hemodialysis and peritoneal dialysis (PD), with posttransplant outcomes.

**Data Sources:**

MEDLINE, Embase, PubMed, Cochrane Library, Scopus, CINAHL, and gray literature were searched from inception to March 18, 2022 (updated to April 1, 2022), for relevant studies and with no language restrictions.

**Study Selection:**

Randomized clinical trials and nonrandomized observational (case-control and cohort) studies that investigated the association between pretransplant dialysis modality and posttransplant outcomes regardless of age or donor sources (living or deceased) were abstracted independently by 2 reviewers.

**Data Extraction and Synthesis:**

Following Preferred Reporting Items for Systematic Reviews and Meta-analyses and Meta-analysis of Observational Studies in Epidemiology reporting guidelines, 2 reviewers independently extracted relevant information using a standardized approach. Random-effects meta-analysis was used to estimate pooled adjusted hazard ratio (HR) or odds ratio and 95% CI.

**Main Outcomes and Measures:**

Primary outcomes included all-cause mortality, overall graft failure, death-censored graft failure, and delayed graft function. Secondary outcomes included acute rejection, graft vessel thrombosis, oliguria, de novo heart failure, and new-onset diabetes after transplant.

**Results:**

The study analyzed 26 nonrandomized studies (1 case-control and 25 cohort), including 269 715 patients (mean recipient age range, 14.5-67.0 years; reported proportions of female individuals, 29.4%-66.9%) whose outcomes associated with pretransplant hemodialysis vs pretransplant PD were compared. No significant difference, with very low certainty of evidence, was observed between pretransplant PD and all-cause mortality (13 studies; n = 221 815; HR, 0.92 [95% CI, 0.84-1.01]; *P* = .08) as well as death-censored graft failure (5 studies; n = 96 439; HR, 0.98 [95% CI, 0.85-1.14]; *P* = .81). However, pretransplant PD was associated with a lower risk for overall graft failure (10 studies; n = 209 287; HR, 0.96 [95% CI, 0.92-0.99]; *P* = .02; very low certainty of evidence) and delayed graft function (6 studies; n = 47 118; odds ratio, 0.73 [95% CI, 0.70-0.76]; *P* < .001; low certainty of evidence). Secondary outcomes were inconclusive due to few studies with available data.

**Conclusions and Relevance:**

Results of the study suggest that pretransplant PD is a preferred dialysis modality option during the transition to kidney transplant. Future studies are warranted to address shared decision-making between health care professionals, patients, and caregivers as well as patient preferences.

## Key Points

**Question:**

What benefits and disadvantages are associated with pretransplant dialysis modalities for posttransplant outcomes in patients with end-stage kidney disease?

**Findings:**

In this systematic review and meta-analysis of 26 nonrandomized studies with 269 715 patients, individuals who underwent peritoneal dialysis had a significantly lower risk for delayed graft function and overall graft failure than those who were treated with hemodialysis. No significant differences were observed in the pretransplant dialysis modality comparisons for all-cause mortality and death-censored graft failure.

**Meaning:**

Findings of the study suggest that peritoneal dialysis during the transition to kidney transplant can be recommended as a preferred dialysis modality and that future studies are needed to examine shared decision-making and patient preference.

## Teaser

This systematic review and meta-analysis describes the risks and outcomes associated with hemodialysis and peritoneal dialysis for patients with end-stage kidney disease.

## Introduction

Over the past several decades, the number of patients with end-stage kidney disease (ESKD) requiring kidney replacement therapy has increased substantially.^1^ Ultimately, kidney transplant is the preferred treatment option for patients with ESKD because it offers substantial benefits in terms of improved life expectancy and health-related quality of life as well as reduced health care costs.^2^ In the US, only 2.9% of new patients with ESKD receive a preemptive kidney transplant; however, most patients with ESKD are initially treated with in-center hemodialysis (HD), with 10.9% undergoing peritoneal dialysis (PD).^3^ Despite improvements in the treatment practice and outcomes of patients undergoing PD, some concerns remain about the high rate of PD-related infections, technique failure, and physician-specific factors (eg, inadequate training and lack of experience) that limit the use of PD.^4,5^

Most patients with ESKD are not able to receive a preemptive kidney transplant or timely transplant due to the lack of suitable kidney donors, late referral to nephrology, or ongoing health and/or financial barriers. In these circumstances, pretransplant dialysis has become a treatment option during the transition to kidney transplant. However, the benefits and disadvantages of different pretransplant dialysis modalities and posttransplant outcomes remain controversial. Given the challenges in the randomization of dialysis modality, numerous observational studies have attempted to compare the association of pretransplant dialysis modality with posttransplant outcomes^6,7,8,9,10^; however, the findings of these studies were inconclusive. There remains clinical equipoise regarding the association of the pretransplant dialysis modality with short-term (ie, graft function and complications) and long-term (ie, patient survival and cardiovascular events) posttransplant outcomes.

Previous systematic reviews have shown that PD is associated with substantial improvement in patient survival and delayed graft function (DGF) compared with HD treatment.^11,12^ However, there are several compelling reasons to reevaluate these findings in light of contemporary evidence. The key limitations of existing systematic reviews include the following: summary results being based on the synthesis of a combination of unadjusted and adjusted effect estimates, which may be subject to residual confounding; studies being restricted to nondiverse ESKD populations; and most studies excluding multiple recent studies in the area.^11,12^ To address this knowledge gap and facilitate a better understanding, we performed a systematic review and meta-analysis to summarize the available evidence of the association of different pretransplant dialysis modalities, including HD and PD, with posttransplant outcomes.

## Methods

The prespecified protocol for this systematic review and meta-analysis was prospectively registered in PROSPERO (CRD42018083917). We followed the Preferred Reporting Items for Systematic Reviews and Meta-analyses (PRISMA) reporting guideline^13^ and the Meta-analysis of Observational Studies in Epidemiology (MOOSE) reporting guideline.^14^

### Systematic Literature Search and Study Selection

Electronic databases, including MEDLINE, Embase, PubMed, Cochrane Library, Scopus, and CINAHL, were searched from inception to March 18, 2022, without language restrictions (eTable 1 in the Supplement). To identify all relevant articles, we supplemented the search with gray literature from Google Scholar, key scientific nephrology and transplant meetings, and preprint reports. Moreover, the manual search of relevant publications as well as key nephrology and transplant journals was extended to April 1, 2022.

Details of the selection criteria are described in eTable 2 in the Supplement. Briefly, both randomized clinical trials and observational (case-control and cohort) studies were included if they (1) investigated the association between dialysis modality and outcomes among kidney transplant recipients (KTRs) regardless of age and donor source (living or deceased); (2) consisted of 2 or more groups undergoing a dialysis modality, including in-center HD, home HD, automated PD, and continuous ambulatory PD; and (3) reported the outcomes of interest or provide sufficient information to calculate the effect estimate. Studies were excluded if they reported only unadjusted effect estimates; involved participants who received a combination of HD and PD treatments; or were case report or case series, cross-sectional studies, reviews, or had no control group. For studies that had overlapping participants or study periods, relevant information was combined, and the study with the most detailed information was considered.

### Outcomes, Data Extraction, and Risk of Bias Assessment

The primary outcomes of interest were all-cause mortality, overall graft failure, death-censored graft failure, and DGF. The secondary outcomes were acute rejection, graft vessel thrombosis, oliguria (not producing urine in the first 24 hours), de novo heart failure, and new-onset diabetes after transplant. Additional outcomes included changes in estimated glomerular filtration rate, hospitalization, retransplant, reentry of maintenance dialysis, and health-related quality of life.

Two of us (T.N. and S.N.) independently extracted the prespecified data using a standardized approach to obtain the following relevant information: study characteristics (ie, study design, study population [KTRs or patients who underwent simultaneous pancreas-kidney transplantation], sample size, setting, study period, publication date, and analysis method), recipient characteristics (ie, recipient age, sex, body mass index, race and ethnicity [which were either self-selected from a list of categories or reported in the data sources], etiology of ESKD, comorbidities, dialysis vintage, and laboratory results), donor and peritransplant characteristics (ie, donor age, donor type, cold ischemia time, and panel reactive antibody), specific exposure and control groups, and outcomes of interest (dialysis modality, definitions, and outcome measurements). The corresponding author of the potentially eligible article was contacted if clarification of studies with unclear or incomplete information was needed.

The risk of bias of each included study was independently assessed by 2 of us (T.N. and S.N.) using the Cochrane risk-of-bias tool for randomized trials^15^ and the Newcastle-Ottawa Scale (NOS; score range: 0-9, with the higher scores indicating the higher quality of the study) for nonrandomized studies.^16^ The overall risk of bias was then categorized as low, high, or of some concern for randomized trials and the highest quality (NOS score ≥8 points) for nonrandomized studies.^17,18^ Any discrepancies in each review step were resolved through consensus discussion.

### Statistical Analysis

All analyses were performed using Stata, version 16.0 (StataCorp LLC). Differences with a 2-tailed *P* < .05 were considered to be statistically significant. To account for potential confounders in nonrandomized studies, we used aggregate risk estimates based on the greatest degree of adjustment for confounding factors as the summary effect estimates for each outcome. To address methodological and statistical heterogeneity between studies, a random-effects model was used to estimate the pooled adjusted hazard ratio (HR) or odds ratio with a corresponding 95% CI as common risk estimates across the included studies.^19^ Furthermore, 95% prediction intervals were calculated for all pooled estimates, which accounted for an estimated range for the true treatment effect in an individual study and the expected uncertainty of the estimate in a new study.^20^ The expected value (E-value), which addresses the robustness of the effect estimates between the pretransplant dialysis modality and posttransplant outcomes to potential residual confounders, was also calculated.^21^

Statistical heterogeneity was evaluated using the Cochran *Q* test using *P* < .05. The degree of inconsistency was assessed on the basis of the *I*^2^ index and τ^2^ statistics as follows: low (*I*^2^ = 25.0%; τ^2^ = 0.01), moderate (*I*^2^ = 50.0%; τ^2^ = 0.06), and high (*I*^2^ = 75.0%; τ^2^ = 0.16).^22^ Funnel plots were visualized for each outcome of interest for which there were sufficient data. Statistical publication bias was investigated using Begg and Egger tests for each specific outcome of interest, with *P* < .10.^23,24^

Preplanned subgroup analyses were performed according to study characteristics, recipient characteristics, and donor and peritransplant characteristics. To address the robustness of the findings, we conducted a set of sensitivity analyses as follows: we restricted analysis to studies that adjusted for key confounding factors (recipient age, donor type, and cold ischemia time), restricted analysis to studies we deemed to be of the highest quality (NOS score ≥8 points), included studies with the directness of effect estimates, excluded studies that were conducted among patients who underwent simultaneous pancreas-kidney transplantation, and included a post hoc analysis using the leave-one-out approach (ie, removing individual studies 1 at a time to assess their role in summary estimates). In addition, a random-effects univariate metaregression was performed to explore the association of prespecified study characteristics, recipient characteristics, and donor and peritransplant characteristics with the meta-analytic estimates.

To interpret evidence findings, 2 of us (T.N. and S.N.) independently appraised evidence certainty using the modified guidance of Grading of Recommendations, Assessment, Development, and Evaluations and the Agency for Healthcare Research and Quality.^25,26^ Evidence certainty was classified as insufficient, very low, low, moderate, or high. Using a contextualized approach to inform clinical interpretation in the context of clinical and methodological viewpoints, we classified the outcome of the dialysis modality as trivial (ie, not substantially different from the comparator), harmful, or beneficial with a particular outcome.

## Results

A total of 26 nonrandomized studies (25 cohort and 1 case-control)^6,7,8,9,10,27,28,29,30,31,32,33,34,35,36,37,38,39,40,41,42,43,44,45,46,47^ with 269 715 patients were included in the present systematic review and meta-analysis. We compared patients with ESKD who underwent pretransplant HD or PD (eFigure 1 in the Supplement). However, no studies that specified other dialysis modalities and no randomized clinical trials were identified. Considering that some included studies had missing data, the proportion of female patients reported ranged from 29.4% to 66.9% (n = 35-107). The mean age ranged from 14.5 to 67.0 years for recipients and 13.8 to 49.2 years for donors.

Most of the studies included KTRs who underwent a deceased-donor kidney transplant, with a mean (SD) cold ischemia time of 8.6 (1.9) hours to 23.9 (6.6) hours (Table 1). The summary NOS score ranged from 6 to 9 points, with 9 studies (34.6%) rated to be of high quality.^6,9,28,29,31,32,34,38,45^ Details of the included studies and their risk-of-bias assessments are provided in eTables 3 and 4 in the Supplement.

**Table 1.** Characteristics of the 26 Included Studies

Source	Study site (design)	Total sample size (PD:HD modality)	Study population	Database used	Study period	Recipient characteristics			Donor characteristics		Cold ischemia time, mean (SD), h	Outcomes reported	NOS score^a^
						Age, mean (SD), y	Female sex, No. (%)	Dialysis vintage, mean (SD), y	Age, mean (SD), y	Donor type: No. (%)			
Pérez Fontán et al,^27^ 1998	Spain (retrospective cohort)	827 (127:700)	Patients with ESKD with extensive use of suboptimal donors^b^	Hospital-based: Hospital Juan Canalejo	Jan 1988-Jul 1997	43.5 (NS)	324 (39.2)	2.7 (NS)	NR	Deceased: 827 (100.0)	NR	Graft vessel thrombosis	7
Bleyer et al,^28^ 1999	US (retrospective cohort)	9291 (NS)	Adult patients with ESKD aged ≥18 y	National registry: UNOS	Apr 1994-Dec 1995	45.2 (13.1)	NS (PD: 42%; HD: 35%)	2.7 (2.7)	NR	Deceased: 9291 (100.0)	21.4 (8.7)	DGF, oliguria^c^	8
Ojo et al,^29^ 1999^d^	US (case-control)	1991 (502:1489)	Adult patients with ESKD aged ≥18 y	National registry: UNOS and USRDS	1990-1996	41.9 (12.7)	1017 (45.7)	2.3 (2.5)	33.1 (16.4)	Living: 424 (19.1)	19.5 (12.7)	Graft vessel thrombosis	8
Van Biesen et al,^30^ 2000	Belgium (retrospective cohort)	119 (40:79)	Adult patients with ESKD aged 18-70 y who were first KTRs	Hospital-based: University Hospital of Gent	Jan 1990 -Dec 1995	42.9 (15.1)	35 (29.4)	NR	NR	Deceased: 119 (100.0)	20.2 (6.9)	DGF	7
Snyder et al,^31^ 2002	US (retrospective cohort)	22 776 (5621:17 155)	Adult patients with ESKD aged ≥18 y	CMS and UNOS Transplant Recipient Registration	1995-1998	18-64 (19 877 [87.3]); ≥65 (2899 [12.7])	10 649 (46.8)	NR	NR	NR	NR	All-cause mortality, overall graft failure, death-censored graft failure, DGF	9
Chalem et al,^32^ 2005	France (retrospective cohort)	3138 (400:2738)	Adult patients with ESKD aged ≥18 y	National information system of the French Transplantation Agency	Jan 1997-Dec 2000	45.9 (12.9)	1122 (35.8)	1.9 (2.7)	40.8 (14.4)	Deceased: 3138 (100.0)	21.4 (8.3)	Overall graft failure	8
Fontana et al,^33^ 2005	Italy (retrospective cohort)	174 (79:95)	Pediatric patients with ESKD who were first KTRs	Hospital-based: S. Martino University	Jun 1987-Sep 2001	14.5 (5.4)	NR	NR	13.8 (10.8)	Deceased: 174 (100.0)	15.2 (3.6)	DGF	7
Goldfarb-Rumyantzev et al,^34^ 2005^e^	US (retrospective cohort)	92 844 (20 240:66 198)	Pediatric and adult patients with ESKD who underwent kidney transplant or SPKT	USRDS	Jan 1990-Dec 2000	43.3 (14.2)	36 859 (39.7)	NR	34.4 (15.5)	Living: 23 025 (24.8)	15.5 (8.7)	All-cause mortality, overall graft failure	8
Resende et al,^35^ 2009	Portugal (retrospective cohort)	421 (47:374)	Adult patients with ESKD aged ≥18 y who were first KTRs	Hospital-based: University Hospital of Santa Maria	May 1989-May 2007	44.4 (12.6)	137 (32.5)	2.8 (1.9)	37.5 (15.4)	Deceased heart-beating donors: 421 (100.0)	19.6 (4.6)	Overall graft failure	6
Courivaud et al,^36^ 2011	France (retrospective cohort)	1896 (332:1564)	Adult patients with ESKD aged ≥18 y without history of diabetes	5 Kidney transplants in France based on university-affiliated medical centers	Jan 1995-Dec 2005	45.5 (13.2)	704 (37.1)	NR	NR	NR	NR	NODAT	6
Madziarska et al,^37^ 2011	Poland (retrospective cohort)	308 (48:260)	Adult patients with ESKD aged ≥18 y without history of diabetes	Hospital-based: Wroclaw Medical University	Jan 2003-Dec 2005	43.9 (12.9)	122 (39.6)	2.1 (2.3)	45.4 (12.3)	Deceased: 308 (100.0)	23.9 (6.6)	NODAT	6
Schwenger et al,^38^ 2011	International (retrospective cohort)	57 315 (11 664: 45 651)	Adults patients with ESKD aged ≥18 y who were first KTRs	Collaborative Transplant Study transplant centers in Europe (86.3%), North America (8.2%), and Australia and New Zealand (5.4%)	1998-2007	49.4 (12.8)	21 358 (37.3)	3.6 (3.2)	46.2 (17.0)	Deceased: 57 315 (100.0)	17.2 (6.8)	All-cause mortality, overall graft failure, death-censored graft failure	8
Sezer et al,^39^ 2011	Turkey (retrospective cohort)	250 (70:180)	Patients with ESKD aged >16 y who were first KTRs	Hospital-based Başkent University School of Medicine	Jan 2000-Dec 2005	36.7 (9.7)	91 (36.4)	1.8 (0.9)	31.0 (NS)	Living: 178 (71.2); deceased: 72 (28.8)	NR	DGF	6
Kramer et al,^40^ 2012	International (retrospective cohort)	29 088 (10 135:18 953)	Adult patients with ESKD aged >20 y who were first KTRs	ERA-EDTA Registry (16 national or regional kidney registries)	Jan 1999-Dec 2008	51.3 (14.9)	10 675 (36.7)	2.4 (1.8)	NR	Living: 4947 (17.0); deceased: 24 141 (83.0)	NR	All-cause mortality, overall graft failure	7
Molnar et al,^6^ 2012	US (retrospective cohort)	14 508 (2092:12 416)	Adult patients with ESKD aged ≥18 y who were first KTRs	Scientific Registry of Transplant Recipients and DaVita data	Jul 2001-Jun 2006	46.5 (14.1)	5721 (39.4)	<2: 5928 (40.9); 2-5: 5265 (36.3); >5: 3315 (22.8)	38.5 (15.0)	Living: 4850 (33.4); deceased: 6964 (48.0); expanded criteria: 2694 (18.6)	13.5 (9.9)	All-cause mortality, death-censored graft failure, DGF	9
López-Oliva et al,^7^ 2014	Spain (retrospective cohort)	236 (118:118)	Adult patients with ESKD aged ≥18 y (58 cases matched by donor)	Hospital-based: University Hospital of La Paz	Dec 1990-Dec 2002	45.5 (12.8)	103 (43.6)	6.6 (4.3)	45.2 (15.6)	Living: 8 (3.4); deceased: 226 (95.8); non–heart-beating donor: 2 (0.8)	17.8 (6.4)	All-cause mortality, overall graft failure	6
Martins et al,^41^ 2015	Portugal (retrospective cohort)	158 (39:119)	Adult patients with ESKD aged ≥18 y with type 1 diabetes who underwent SPKT	Hospital-based: University Hospital of Santo António	May 2000-Dec 2013	34.6 (6.0)	82 (51.9)	2.2 (1.7)	28.2 (10.5)	Deceased: 158 (100.0)	11.2 (4.9)	All-cause mortality	7
Dipalma et al,^42^ 2016	Spain (retrospective cohort)	160 (80:80)	Adult patients with ESKD aged ≥18 y using donor-matched KTR approach	Hospital-based: Hospital Universitario 12 de Octubre	Jan 1990-Dec 2007	44.9 (14.5)	107 (66.9)	2.0 (2.0)	39.2 (18.1)	Deceased: 160 (100.0)	20.5 (4.4)	All-cause mortality, death-censored graft failure	7
Dębska-Ślizień et al,^43^ 2018	Poland (retrospective cohort)	266 (133:133)	Pediatric and adult patients with ESKD aged 12-81 y using donor-matched KTR approach	Hospital-based: Gdansk Transplantation Center	Dec 1994-Dec 2016	46.6 (15.3)	115 (43.2)	2.2 (2.3)	43.3 (14)	Deceased: 266 (100.0)	14.7 (NS)	Graft vessel thrombosis	6
Lin et al,^8^ 2018	Taiwan (retrospective cohort)	1812 (603:1209)	Adult patients with ESKD aged ≥18 y	National Health Insurance	1998-2011	42.6 (12.6)	823 (45.4)	3.1 (2.8)	NR	NR	NR	All-cause mortality, death-censored graft failure	7
Marcacuzco et al,^44^ 2018	Spain (retrospective cohort)	165 (67:98)	Adult patients with ESKD aged ≥18 y with diabetes who underwent SPKT	Hospital-based: Hospital Universitario 12 de Octubre	Mar 1995-Dec 2015	38.9 (7.5)	66 (40.0)	1.8 (1.0)	Median (IQR): 29 (21-35)	NR	8.6 (1.9)	All-cause mortality	6
Balzer et al,^45^ 2020	Germany (retrospective cohort)	2006 (159:1847)	Adult patients with ESKD aged ≥18 y	Hospital-based: Hannover Medical School	Jan 2000-Dec 2014	49.0 (13.1)	771 (38.4)	4.6 (3.0)	49.2 (16.2)	Living: 313 (15.6); deceased: 1693 (84.4)	12.0 (7.1)	All-cause mortality, overall graft failure, acute rejection	9
Scheuermann et al,^46^ 2020	Germany (retrospective cohort)	83 (19:64)	Adult patients with ESKD aged ≥18 y with diabetes (type 1 or 2) who underwent SPKT	Hospital-based: University Hospital of Leipzig	2000-2016	43.5 (9.2)	37 (44.6)	2.4 (1.8)	21.6 (11.0)	Deceased: 83 (100.0)	11.5 (3.2)	All-cause mortality, overall graft failure	7
Lenihan et al,^9^ 2021	US (retrospective cohort)	27 701 (5326:22 375)	Population-based adult patients with ESKD aged ≥18 y who were first KTRs	USRDS	Jan 2005-Sep 2015	47.0 (14.0)	10 878 (39.3)	4.0 (2.7)	39.0 (16.0)	Living: 5881 (21.2); deceased: 17 779 (64.2); expanded criteria: 3868 (14.0)	15.0 (10.6)	De novo heart failure	9
So et al,^10^ 2020	Australia and New Zealand (retrospective cohort)	802 (226:573)	Older adults with ESKD aged ≥65 y who were first KTRs	ANZDATA Registry and National Organ Matching System	Jun 2006-Dec 2016	67.0 (3.3)	271 (33.8)	NR	NR	NR	NR	All-cause mortality	6
Prezelin-Reydit et al,^47^ 2022^f^	France (retrospective cohort)	1380 (289:1067)	Pediatric patients with ESKD who were first KTRs	French organ transplant database and French kidney replacement registry	Jan 1993-Dec 2012	12.5 (5.9)	508 (34.3)	1.2 (1.0)	17.0 (11.9)	Living: 150 (10.1); deceased: 1330 (89.9)	18.2 (7.0)	Overall graft failure	7

Abbreviations: ANZDATA, Australia and New Zealand Dialysis and Transplant; CMS, Centers for Medicare & Medicaid Services; DGF, delayed graft function; ERA-EDTA, European Renal Association–European Dialysis and Transplant Association; ESKD, end-stage kidney disease; HD, hemodialysis; KTR, kidney transplant recipient; NODAT, new-onset diabetes after transplant; NOS, Newcastle-Ottawa Scale; NR, not reported; NS, not specified; PD, peritoneal dialysis; SPKT, simultaneous pancreas-kidney transplantation; UNOS, United Network for Organ Sharing; USRDS, US Renal Data System.

^a^ The NOS scores ranged from 0 to 9, with the higher scores indicating the overall high quality of the study.

^b^ Suboptimal donors included non–heart-beating donors (12.6%), children 5 years or younger (5.4%), and adults older than 60 years (9.1%).

^c^ Not producing urine in the first 24 hours.

^d^ Based on the whole sample (n = 743 cases; n = 1480 control individuals).

^e^ Based on the whole cohort (n = 92 844).

^f^ Based on nonpreemptive kidney transplant cohort.

### Primary and Secondary Outcomes 

**Figure 1.**
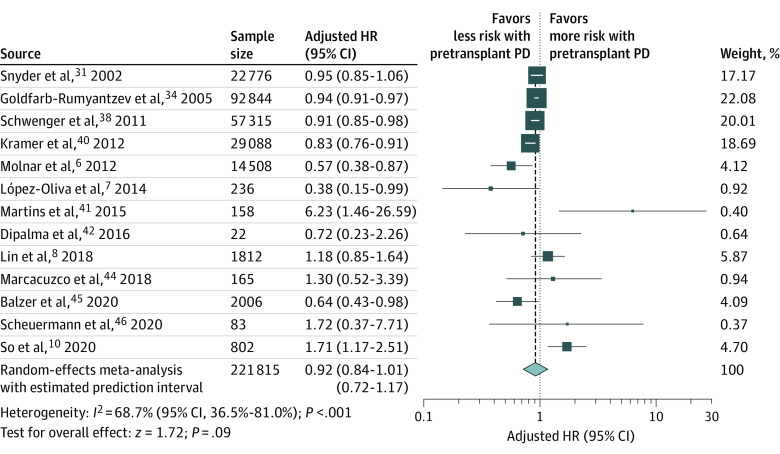
Meta-analysis of Pretransplant Dialysis Modality and the Risk of All-Cause Mortality HR indicates hazard ratio; PD, peritoneal dialysis. The size of the boxes indicates weight of the study in proportion to the pooled estimate, error bars represent 95% CIs, the size of the diamond represents the overall pooled effects, and the width of the diamond represents the 95% CI of the point estimate of the pooled effect.

**Figure 2.**
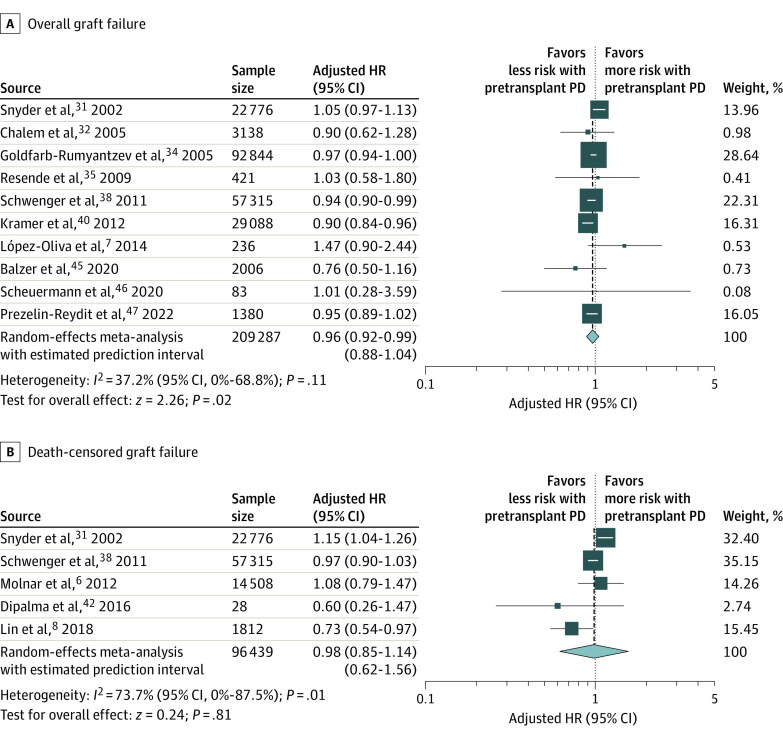
Meta-analysis of Pretransplant Dialysis Modality and the Risk of Overall Graft Failure and Death-Censored Graft Failure HR indicates hazard ratio; PD, peritoneal dialysis. The size of the boxes indicates weight of the study in proportion to the pooled estimate, error bars represent 95% CIs, the size of the diamond represents the overall pooled effects, and the width of the diamond represents the 95% CI of the point estimate of the pooled effect.

**Figure 3.**
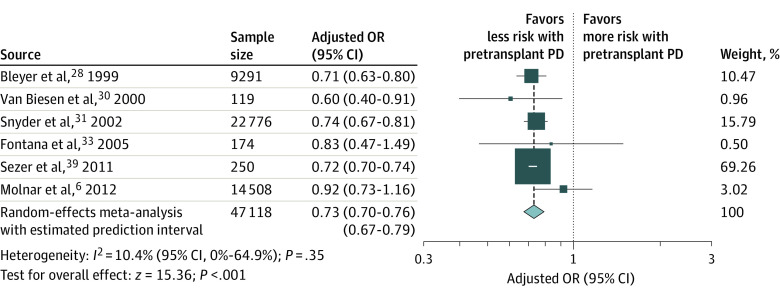
Meta-analysis of Pretransplant Dialysis Modality and the Risk of Delayed Graft Function OR indicates odds ratio; PD, peritoneal dialysis. The size of the boxes indicates weight of the study in proportion to the pooled estimate, error bars represent 95% CIs, the size of the diamond represents the overall pooled effects, and the width of the diamond represents the 95% CI of the point estimate of the pooled effect.

Among patients with ESKD who underwent pretransplant PD compared with HD, there was no statistical difference in terms of all-cause mortality (13 studies^6,7,8,10,31,34,38,40,41,42,44,45,46^; n = 221 815; HR, 0.92 [95% CI, 0.84-1.01]; *P* = .08) (Figure 1) and death-censored graft failure (5 studies^6,8,31,38,42^; n = 96 439; HR, 0.98 [95% CI, 0.85-1.14]; *P* = .81) (Figure 2). However, pretransplant PD revealed a small outcome, with a significantly lower risk for overall graft failure (10 studies^7,31,32,34,35,38,40,45,46,47^; n = 209 287; HR, 0.96 [95% CI, 0.92-0.99]; *P* = .02) (Figure 2). Moreover, pretransplant PD was associated with a decreased risk for DGF (6 studies^6,28,30,31,33,39^; n = 47 118; OR, 0.73 [95% CI, 0.70-0.76]; *P* < .001) (Figure 3). A summary of pretransplant dialysis modality and posttransplant outcomes is presented in Table 2.

**Table 2.** Summary of Findings and Strength of Evidence

Kidney transplant outcomes	No. of included studies (sample size)	Effect estimate, OR or HR (95% CI)	*P* value	E-value for point estimate (95% CI upper limit)	95% Prediction interval	Heterogeneity				Strength of evidence (outcome classification)
						*Q* statistic	*P* value	*I*^2^ index (95% CI), %	τ^2^	
Primary outcomes										
All-cause mortality	13 (n = 221 815)	HR: 0.92 (0.84-1.01)	.08	1.388 (1.000)	0.72-1.17	38.37	<.001	68.7 (36.5-81.0)	0.010	Very low (trivial)
Overall graft failure	10 (n = 209 287)	HR: 0.96 (0.92-0.99)	.02	1.254 (1.084)	0.88-1.04	14.34	.11	37.2 (0.0-68.8)	0.001	Very low (beneficial with PD)
Death-censored graft failure	5 (n = 96 439)	HR: 0.98 (0.85-1.14)	.81	1.155 (1.000)	0.62-1.56	15.23	.01	73.7 (0.0-87.5)	0.016	Very low (trivial)
Delayed graft function	6 (n = 47 118)	OR: 0.73 (0.70-0.76)	<.001	2.098 (1.976)	0.67-0.79	5.58	.35	10.4 (0.0-64.9)	<0.001	Low (beneficial with PD)
Secondary outcomes										
Acute rejection	1 (n = 2006)	OR: 0.70 (0.51-0.97)	.03	2.211 (1.230)	NA	NA	NA	NA	NA	Insufficient data
Graft vessel thrombosis	3 (n = 3084)	OR: 1.35 (0.50-3.65)	.55	2.037 (1.000)	1.00 × 10^−5^ to 1.23 × 10^5^	7.28	.03	72.5 (0.0-89.7)	0.550	Very low (trivial)
Oliguria (not producing urine in the first 24 h)	1 (n = 9291)	OR: 0.74 (0.62-0.87)	<.001	2.057 (1.557)	NA	NA	NA	NA	NA	Insufficient data
De novo heart failure	1 (n = 27 701)	OR: 0.84 (0.78-0.91)	<.001	1.667 (1.429)	NA	NA	NA	NA	NA	Insufficient data
NODAT	2 (n = 2204)	OR: 1.57 (0.56-4.45)	.39	2.522 (1.000)	NA	5.48	.02	81.8 (NA)	0.463	Very low (trivial)

Abbreviations: HR, hazard ratio; NA, not applicable; NODAT, new-onset diabetes after transplant; OR, odds ratio; PD, peritoneal dialysis.

The summary effect sizes for secondary outcomes revealed that the dialysis modalities did not differ in terms of graft vessel thrombosis and new-onset diabetes after transplant. However, it was not possible to compare the outcomes of the pretransplant dialysis modality and other secondary outcomes due to the limited number of included studies (Table 2). Meanwhile, no study reported additional outcomes of interest.

### Subgroup Analysis, Sensitivity Analysis, Metaregression, and Publication Bias

Several a priori subgroup analyses of KTR characteristics could not be performed due to the limited number of included studies. However, the association between pretransplant PD and all-cause mortality was observed when subgroup analyses were based on studies published before 2015,^6,31,34,38,40,41^ restricted to studies with a sample size greater than 1000 patients,^6,8,31,34,38,40,45^ or limited to only pediatric or mixed-case populations.^34^ Regarding overall graft failure, no association was observed among studies with adult cases,^7,31,32,35,38,40,45,46^ mixed or unspecified donor type,^7,31,34,40,45,47^ publication date before 2015^7,31,32,34,35,38,40^ or from 2015 to 2022,^45,46,47^ and single-center design.^7,35,45,46^ Nevertheless, subgroup analyses did not reveal any association between pretransplant PD and death-censored graft failure or DGF (eTable 5 in the Supplement).

For sensitivity analyses, the results for death-censored graft failure and DGF were robust and similar to the main findings. Nevertheless, the association between dialysis modality and all-cause mortality and overall graft failure appeared to be sensitive and inconsistent based on a set of sensitivity analyses (eTables 6, 7, 8, 9, and 10 in the Supplement). For example, after restricted analysis, there was no association in studies that adjusted for key confounding factors or were deemed to be of the highest quality (NOS score ≥8 points) or studies with the directness of effect estimates for overall graft failure.

According to univariate metaregression analyses, study characteristics (setting and location: HR, 1.58 [95% CI, 1.02-2.44]; *P* = .04) were associated with the higher risk of all-cause mortality, recipient characteristics (proportion of female: HR, 1.01 [95% CI, 1.00-1.02], *P* = .02; diabetes status: HR, 1.00 [95% CI, 1.00-1.01], *P* = .03) were associated with the higher risk of overall graft failure, and risk of bias by NOS score (HR, 1.23; 95% CI, 1.05-1.44; *P* = .03) was associated with the higher risk of death-censored graft failure (eTable 11 in the Supplement). In contrast, dialysis vintage was associated with a lower risk of all-cause mortality (HR, 0.82; 95% CI, 0.70-0.98; *P* = .03). Moreover, publication bias was not identified for any outcome of interest (all *P* > .10 in Begg and Egger tests) (eTable 12 in the Supplement). The funnel plots for each outcome are presented in eFigure 2 in the Supplement.

### Certainty of Evidence

Given the evidence-based synthesis, pretransplant PD, compared with HD, revealed a benefit with a low strength of evidence for DGF, based on evidence certainty and robustness of the effect estimates in terms of the prediction interval (0.67-0.79) and E-value (2.098; 95% CI upper limit, 1.976) (Table 2). Although pretransplant PD was also beneficial for overall graft failure, it was downgraded to very low strength of evidence because the prediction interval (0.88-1.04) revealed evidence of uncertainty (Table 2). Other outcomes were graded as having very low or insufficient certainty of evidence and classified as being trivial (Table 2). Details of evidence synthesis for each outcome are provided in eTable 13 in the Supplement.

## Discussion

We summarized evidence from 26 nonrandomized studies that evaluated the association between pretransplant dialysis modality and posttransplant outcomes. Individuals who underwent PD had a significantly lower risk for DGF and overall graft failure, with very low to low certainty of evidence, than those who were treated with HD. No significant differences were observed in the pretransplant dialysis modality comparisons for all-cause mortality, death-censored graft failure, graft vessel thrombosis, and new-onset diabetes after transplant. However, the association of the pretransplant dialysis modality with acute rejection, oliguria, and de novo heart failure was inconclusive due to insufficient data.

This systematic review and meta-analysis included large sample sizes and up-to-date and expanded evidence of the association of pretransplant dialysis modality with posttransplant outcomes. These studies included diverse populations of KTRs (ie, children, adults, and patients who underwent simultaneous pancreas-kidney transplantation). We performed a rigorous and comprehensive systematic review using adjusted risk estimates to account for potential confounders. This study had important methodological differences from previous meta-analyses.^11,12^ We expanded the risk estimates across comprehensively relevant posttransplant outcomes. Only studies with adjusted risk estimates were included and pooled for meta-analysis. Using a noncontemporary studies cohort (based on data before 2014) and mixed unadjusted and adjusted risk estimates, 2 meta-analyses by Tang et al^12^ (12 nonrandomized studies) and Joachim et al^11^ (16 nonrandomized studies) found that pretransplant PD was associated with a significantly lower risk for DGF (odds ratio, 0.50-0.67) than HD, which was consistent with the findings of the present study. Compared with HD, pretransplant PD was associated with better long-term patient survival (pooled HR, 0.86-0.89) in 2 studies.^11,12^ In contrast, we did not observe any association of the pretransplant dialysis modality with all-cause mortality. Apart from the different approaches to statistical analysis, we postulate that this finding was possible because treatments and techniques for dialysis care have improved over time under modern sophisticated transplant care and immunosuppressive regimens. Nevertheless, further studies are required to clarify this outcome.

The pathophysiological processes and mechanisms that explain the outcome of different dialysis modalities in the short and long terms during transition to transplant and after transplant are not well established. Previous studies have suggested that immunologic differences may be factors in posttransplant outcomes.^48,49^ Compared with HD, pretransplant PD generally provides better native and residual kidney function preservation, thereby partly explaining the posttransplant outcomes, including DGF and survival.^4,50,51,52^ Pretransplant PD also provides more favorable perioperative fluid balance than HD. Meanwhile, pretransplant HD may be associated with volume depletion or a higher proinflammatory state resulting in inadequate perfusion of the allograft and tubular necrosis.^4,28^ In addition, long-term pretransplant HD supplemented further risk for cardiovascular events through intermittent and nonphysiological volume shifts, which may be a factor in adverse cardiovascular outcomes.^53^ This finding is supported by a large cohort study from the US that reported an association between pretransplant HD and a higher risk for de novo posttransplant heart failure (HR, 1.19; 95% CI, 1.09-1.29).^9^

### Implications for Practice and Future Research

Although a comparison of randomized clinical trials on PD and HD is currently not available and challenging to conduct, we believe that pretransplant PD is a reasonable alternative option and should not be discouraged during the transition to kidney transplant, owing to the limited number of living donors. With respect to global disparities in organ transplant practices and patterns, we encourage health care professionals to adopt shared decision-making with all candidate KTRs in the nephrology practice and their caregivers for the most appropriate goal-directed dialysis program. To form a judgment on patient preferences, health care professionals should inform candidate KTRs about the advantages and disadvantages of various dialysis modalities for both short- and long-term posttransplant outcomes. Furthermore, future studies comparing dialysis modalities need to incorporate current dialysis treatment options, such as home dialysis, and need to be restricted to candidate KTRs who are considered to be eligible for either HD or PD, which reaffirms the association of dialysis modality with posttransplant outcomes.

### Limitations

This systematic review and meta-analysis has several potential limitations. First, because the identified studies were nonrandomized, the pooled estimates were subject to confounding by indications or contraindications. Thus, causal associations cannot be established. Second, given that the findings were based on adjusted risk estimates, we could not entirely exclude residual confounders for the effect estimates based on the varying degree of confounder adjustment between the studies. Only 9 of the included studies^6,9,27,28,31,32,38,45,47^ addressed the key factors (recipient age, donor type, and cold ischemia time) in the model of analysis. Furthermore, based on the prediction interval and E-value, the uncertainty of the findings and potential residual confounders remained. As a result, we downgraded and rated the strength of the evidence to be low or very low. Third, the quality of the included studies varied; however, post hoc sensitivity analysis, including studies with the highest quality, demonstrated no significant difference from the main findings, except for all-cause mortality. Fourth, differences were observed in the study population, protocol and policy for kidney transplant, and treatment patterns across settings, which could explain the heterogeneity of the pooled effect estimates. Fifth, information bias may exist because most of the included studies obtained data from routine, administrative databases^6,7,8,9,10,28,29,31,32,34,36,37,38,40,41,47^; however, most studies identified outcomes of interest through the *International Classification of Diseases* or national transplantation registries.^6,8,9,10,28,29,31,32,34,36,38,40,47^ Sixth, despite the large sample size and inclusion of recent evidence, some relevant outcomes (eg, early vs late graft failure) were limited. Moreover, other dialysis treatment options, particularly home HD, as well as the specific treatment modalities for in-center HD (ie, conventional, short daily) and PD (ie, automated PD and continuous ambulatory PD) were not available. Seventh, we lacked information regarding baseline candidate health status and donor-specific and peritransplant characteristics, such as residual kidney function, comorbid conditions, dialysis vintage, and immunosuppressive regimens. Therefore, the effect estimates for these subpopulations could not be established.

## Conclusions

Results of this systematic review and meta-analysis suggest that PD during the transition to kidney transplant can be recommended as a preferred dialysis modality for patients with ESKD. However, the certainty of the evidence was very low to low. Future studies are warranted, particularly collaborative prospective studies or pragmatic trials with diverse KTR populations, to examine shared decision-making between health care professionals, patients, and caregivers and the preferences of patients.

## Notes

Supplement. **eTable 1.** Systematic Review Search Strategy **eTable 2.** The PICOTS Format: Study Inclusion/Exclusion Criteria **eTable 3.** Characteristics of Study Participants Included in the Meta-Analysis **eTable 4.** Risk of Bias Assessment of Included Studies by the NOS **eTable 5.** Subgroup Analysis of Primary Outcomes **eTable 6.** Sensitivity Analysis: Restricting the Analysis to Studies That Adjusted for Key Confounding Factors **eTable 7.** Sensitivity Analysis: Restricting the Analysis to Studies Judged to Be of the Highest Quality (NOS ≥8 Points) **eTable 8.** Sensitivity Analysis: Including the Analysis of Studies With the Directness of Effect Estimates **eTable 9.** Sensitivity Analysis: Excluding Studies That Were Conducted Among SPKT Patients **eTable 10.** Sensitivity Analysis: Post-Hoc Analysis Using the “Leave-One-Out” Approach **eTable 11.** Meta-Regression of Primary Outcomes **eTable 12.** Publication Bias **eTable 13.** Quality of Evidence Synthesis and GRADE Evidence Profile of Outcomes **eFigure 1.** PRISMA Flow Diagram of the Literature Search and Selection **eFigure 2.** Funnel Plot of Included Studies in the Meta-Analysis
eReferences
