# Supplementary material for: Association Between Pretransplant Dialysis Modality and Kidney Transplant Outcomes: A Systematic Review and Meta-analysis
Source: JAMA Netw Open. 2022 Oct 20;5(10):e2237580. doi: 10.1001/jamanetworkopen.2022.37580 (PMC9585427; doi:10.1001/jamanetworkopen.2022.37580)
Supplement: Supplement. — eTable 1. Systematic Review Search Strategy eTable 2. The PICOTS Format: Study Inclusion/Exclusion Criteria eTable 3. Characteristics of Study Participants Included in the Meta-Analysis eTable 4. Risk of Bias Assessment of Included Studies by the NOS eTable 5. Subgroup Analysis of Primary Outcomes eTable 6. Sensitivity Analysis: Restricting the Analysis to Studies That Adjusted for Key Confounding Factors eTable 7. Sensitivity Analysis: Restricting the Analysis to Studies Judged to Be of the Highest Quality (NOS ≥8 Points) eTable 8. Sensitivity Analysis: Including the Analysis of Studies With the Directness of Effect Estimates eTable 9. Sensitivity Analysis: Excluding Studies That Were Conducted Among SPKT Patients eTable 10. Sensitivity Analysis: Post-Hoc Analysis Using the “Leave-One-Out” Approach eTable 11. Meta-Regression of Primary Outcomes eTable 12. Publication Bias eTable 13. Quality of Evidence Synthesis and GRADE Evidence Profile of Outcomes eFigure 1. PRISMA Flow Diagram of the Literature Search and Selection eFigure 2. Funnel Plot of Included Studies in the Meta-Analysis eReferences [file jamanetwopen-e2237580-s001.pdf]

Supplemental Online Content

Ngamvichchukorn T, Ruengorn C, Noppakun K, et al. Association between pretransplant dialysis modality and kidney transplant outcomes: a systematic review and meta-analysis. *JAMA Netw Open*. 2022;5(10):e2237580. doi:10.1001/jamanetworkopen.2022.37580

eTable 1. Systematic Review Search Strategy

eTable 2. The PICOTS Format: Study Inclusion/Exclusion Criteria

eTable 3. Characteristics of Study Participants Included in the Meta-Analysis

eTable 4. Risk of Bias Assessment of Included Studies by the NOS

eTable 5. Subgroup Analysis of Primary Outcomes

eTable 6. Sensitivity Analysis: Restricting the Analysis to Studies That Adjusted for Key Confounding Factors

eTable 7. Sensitivity Analysis: Restricting the Analysis to Studies Judged to Be of the Highest Quality (NOS ≥8 Points)

eTable 8. Sensitivity Analysis: Including the Analysis of Studies With the Directness of Effect Estimates

eTable 9. Sensitivity Analysis: Excluding Studies That Were Conducted Among SPKT Patients

eTable 10. Sensitivity Analysis: Post-Hoc Analysis Using the "Leave-One-Out" Approach

eTable 11. Meta-Regression of Primary Outcomes

eTable 12. Publication Bias

eTable 13. Quality of Evidence Synthesis and GRADE Evidence Profile of Outcomes

eFigure 1. PRISMA Flow Diagram of the Literature Search and Selection

eFigure 2. Funnel Plot of Included Studies in the Meta-Analysis

eReferences

This supplemental material has been provided by the authors to give readers additional information about their work.

© 2022 Ngamvichchukorn T et al. *JAMA Network Open.*

eTable 1. Systematic Review Search Strategy

OVID: Medline (From Inception to March 18, 2022)		
**Search**	**Query**	**Items Found**
#1	exp Renal Dialysis/	121,201
#2	((end stage or endstage) adj (kidney or renal or dialysis)).tw,kw,rn.	46,448
#3	((kidney or renal) adj dialy*).tw,kw,rn.	1,563
#4	(dialy* adj (patient* or therapy or modalit*)).tw,kw,rn.	23,650
#5	exp H$emodialysis/	121,201
#6	(h$emodialy* or h$emofilt* or intradialy*).tw,kw,rn.	74,139
#7	(in-center or incenter or nocturnal or home) adj3 (h$emodialy* or dialy*).tw,kw,rn.	2,840
#8	exp Peritoneal Dialysis/	27,436
#9	*dialysis, peritoneal/	14,577
#10	(tenckhoff* automated or continuous or ambulatory) adj3 (peritoneal or dialy*).tw,kw,rn.	8,029
#11	(peritoneal dialysis or pd or capd or ccpd or apd or ipd or nipd or tpd).tw,kw,rn.	200,177
#12	or/1-11	347,979
#13	exp Kidney Transplantation/	101,600
#14	(kidney or renal) adj (transplant* or candidate* or organ or nephrop* or wait list* or recipient*).tw,kw,rn.	84,690
#15	or/13-14	119,517
#16	12 and 15	20,596
#17	((graft or acute or delayed) adj (function or rejection or failure or survival)).tw,kw,rn.	58,512
#18	(outcome or death or mortality or survival or cardiovascular or glomerular filtration or hospitali$ation or infection* or re-transplant* or vascular thrombosis or costs or health-related quality of life or hrqol or qol or physical function or psychological or mental health or psychosocial or patient-reported outcomes).tw,kw,rn.	5,458,065
#19	or/17-18	5,476,647
#20	16 and 19	11,100
#21	Randomized Controlled Trials as Topic/	153,578
#22	random allocation/	106,741
#23	Controlled Clinical Trials as Topic/	5,625
#24	control groups/	1,817
#25	clinical trials as topic/ or clinical trials, phase i as topic/ or clinical trials, phase ii as topic/ or clinical trials, phase iii as topic/ or clinical trials, phase iv as topic/	218,772
#26	randomized controlled trial.pt.	561,679

© 2022 Ngamvichchukorn T et al. *JAMA Network Open.*

#27	controlled clinical trial.pt.	94,744
#28	(clinical trial or clinical trial phase i or clinical trial phase ii or clinical trial phase iii or clinical trial phase iv).pt.	591,229
#29	(random$ or RCT or RCTs).tw,kw.	1,309,697
#30	(randomi?ed or randomly or RCT$1 or placebo$).tw,kw.	1,105,268
#31	((singl$ or doubl$ or trebl$ or tripl$) adj5 (mask$ or blind$ or dumm$)).tw,kw.	190,122
#32	(controlled adj5 (trial$ or stud$)).tw,kw.	441,956
#33	(clinical$ adj5 trial$).tw,kw.	473,067
#34	((control or treatment or experiment$ or intervention) adj5 (group$ or subject$ or patient$)).tw,kw.	1,690,649
#35	(quasi-random$ or quasi random$ or pseudo-random$ or pseudo random$).tw,kw.	6,364
#36	((control or experiment$ or conservative) adj5 (treatment or therapy or procedure or manage$)).tw,kw.	6,364
#37	trial.ti.	239,520
#38	(assign$ or allocat$).tw.	486,508
#39	or/21-38	3,899,049
#40	20 and 39	2,877
#41	(nRCT or nRCTs or non-RCT?).tw,kw.	1,200
#42	(control$ adj2 stud$3).tw,kw.	259,374
#43	control group/	1,817
#44	(control$ adj2 group$1).tw,kw.	569,469
#45	exp comparative study/	1,910,498
#46	((comparative or comparison) adj (study or studies)).tw,kw.	120,532
#47	exp cohort study/	2,313,394
#48	(cohort$ adj2 stud$3).tw,kw.	292,767
#49	exp case control study/	1,296,808
#50	((case-control$ or case-based or case-comparison) adj (study or studies)).tw,kw.	120,692
#51	or/41-50	4,743,817
#52	20 and 51	4,544
#53	40 or 52	6,106
#54	(comment or editorial or interview or letter or news or newspaper article).pt.	2,285,371
#55	53 not 54	6,073
#56	limit 55 to human	5,709

© 2022 Ngamvichchukorn T et al. *JAMA Network Open.*

eTable 1. Systematic Review Search Strategy (Continued)

**Embase via Elsevier 1966 to March 18, 2022)**		
**Search**	**Query**	**Items Found**
#1	Renal Dialysis/exp AND [embase]/lim	98529
#2	((‘end stage’:ti,ab OR endstage:ti,ab) AND (kidney:ti,ab OR renal:ti,ab OR dialysis:ti,ab)) AND [embase]/lim	68032
#3	((kidney:ti,ab OR renal:ti,ab) AND dialy*:ti,ab) AND [embase]/lim	81645
#4	(dialy*:ti,ab AND (patient*:ti,ab OR therapy:ti,ab OR modalit*:ti,ab)) AND [embase]/lim	119886
#5	H$emodialysis/exp AND [embase]/lim	153695
#6	(h$emodialy*:ti,ab OR h$emofilt*:ti,ab OR intradialy*:ti,ab) AND [embase]/lim	109132
#7	((‘in-center’:ti,ab OR incenter:ti,ab OR nocturnal:ti,ab OR home:ti,ab) AND (h$emodialy*:ti,ab OR dialy*:ti,ab)) AND [embase]/lim	7407
#8	Peritoneal Dialysis/exp AND [embase]/lim	40432
#9	((tenckhoff*:ti,ab OR automated:ti,ab OR continuous:ti,ab OR ambulatory:ti,ab) AND (peritoneal:ti,ab OR dialy*:ti,ab)) AND [embase]/lim	18689
#10	#1 OR #2 OR #3 OR #4 OR #5 OR #6 OR #7 OR #8 OR #9	266869
#11	‘Kidney Transplantation’/exp AND [embase]/lim	149971
#12	((kidney:ti,ab OR renal:ti,ab) AND (transplant*:ti,ab OR candidate*:ti,ab OR organ:ti,ab OR nephrop*:ti,ab OR ‘wait list*’:ti,ab OR recipient*:ti,ab)) AND [embase]/lim	278066
#13	#11 OR #12	305349
#14	#10 AND #13	66373
#15	((graft:ti,ab OR acute:ti,ab OR delayed:ti,ab) AND (function:ti,ab OR rejection:ti,ab OR failure:ti,ab OR survival:ti,ab)) AND [embase]/lim	638877
#16	(death:ti,ab OR mortality:ti,ab OR survival:ti,ab OR cardiovascular:ti,ab OR ‘glomerular filtration’:ti,ab OR hospitali$ation:ti,ab OR infection*:ti,ab OR retransplant*:ti,ab OR ‘vascular thrombosis’:ti,ab OR cost*:ti,ab OR ‘health related quality of life’:ti,ab OR hrqol:ti,ab OR qol:ti,ab OR ‘physical function’:ti,ab OR psychological:ti,ab OR ‘mental health’:ti,ab OR psychosocial:ti,ab OR ‘patient reported outcome*’:ti,ab) AND [embase]/lim	5974471
#17	#15 OR #16	6226363
#18	#14 AND #17	43583
#19	(news:it OR ‘newspaper article’:it OR comment:it OR editorial:it OR interview:it OR letter:it OR review:it OR ‘systematic review’:it OR ‘case report’:it OR ‘case series’:it) AND [embase]/lim	3635619
#20	#18 NOT #19	38891
#21	#20 AND ([article]/lim OR [article in press]/lim OR [conference abstract]/lim OR [conference paper]/lim)	38186

© 2022 Ngamvichchukorn T et al. *JAMA Network Open.*

#22	#21 AND ([child]/lim OR [preschool]/lim OR [school]/lim OR [adolescent]/lim OR [adult]/lim OR [young adult]/lim OR [middle aged]/lim OR [aged]/lim OR [very elderly]/lim)	26383
#23	#22 AND 'article'/it	14428
#24	#22 AND 'article'/it AND ('case control study'/de OR 'clinical article'/de OR 'clinical study'/de OR 'clinical trial'/de OR 'clinical trial topic'/de OR 'cohort analysis'/de OR 'comparative effectiveness'/de OR 'comparative study'/de OR 'controlled clinical trial'/de OR 'controlled clinical trial topic'/de OR 'controlled study'/de OR 'hospital based case control study'/de OR 'intention to treat analysis'/de OR 'major clinical study'/de OR 'observational study'/de OR 'phase 4 clinical trial'/de OR 'phase 4 clinical trial topic'/de OR 'population based case control study'/de OR 'prospective study'/de OR 'randomized controlled trial'/de OR 'randomized controlled trial topic'/de OR 'retrospective study'/de)	12487

© 2022 Ngamvichchukorn T et al. *JAMA Network Open.*

eTable 1. Systematic Review Search Strategy (Continued)

**PubMed (From Inception to March 18, 2022)**		
**Search**	**Query**	**Items Found**
#1	((((renal dialysis[MeSH Terms]) OR (dialysis[MeSH Terms])) OR (peritoneal dialysis, continuous ambulatory[MeSH Terms])) OR (dialysis, peritoneal[MeSH Terms])) OR (hemodialysis[MeSH Terms])	144,031
#2	((((((((intradialy*[Title/Abstract]) OR (hemodialy*[Title/Abstract])) OR (hemofilt*[Title/Abstract])) OR (in-center dialysis[Title/Abstract])) OR (nocturnal dialysis[Title/Abstract])) OR (home dialysis[Title/Abstract])) OR (peritoneal dialysis[Title/Abstract])) OR (automated peritoneal dialysis[Title/Abstract])) OR (continuous ambulatory peritoneal dialysis[Title/Abstract])) OR (CAPD[Title/Abstract])	95,629
#3	#1 OR #2	168,904
#4	(renal transplantation[MeSH Terms]) AND (kidney transplantation[MeSH Terms])	101,598
#5	(((kidney transplant*[Title/Abstract]) OR (renal transplant*[Title/Abstract])) OR (kidney recipient[Title/Abstract])) OR (renal recipient[Title/Abstract])	85,405
#6	#4 OR #5	119,627
#7	#3 AND #6	13,681
#8	(((graft[Title/Abstract]) OR (acute[Title/Abstract])) OR (delayed[Title/Abstract])) AND ((((function[Title/Abstract]) OR (rejection[Title/Abstract])) OR (failure[Title/Abstract])) OR (survival[Title/Abstract]))	447,441
#9	((((((((((((((((death[Title/Abstract]) OR (mortality[Title/Abstract])) OR (survival[Title/Abstract])) OR (cardiovascular[Title/Abstract])) OR (glomerular filtration[Title/Abstract])) OR (hospitalization[Title/Abstract])) OR (infection*[Title/Abstract])) OR (re-transplant*[Title/Abstract])) OR (vascular thrombosis[Title/Abstract])) OR (costs[Title/Abstract])) OR (health-related quality of life[Title/Abstract])) OR (HRQOL[Title/Abstract])) OR (QOL[Title/Abstract])) OR (physical function[Title/Abstract])) OR (psychological[Title/Abstract])) OR (mental health[Title/Abstract])) OR (psychosocial[Title/Abstract])) OR (patient-reported outcomes[Title/Abstract])	4,918,083
#10	#8 OR #9	5,121,717
#11	#7 AND #10	6,748
#12	(((((((Case Reports[Publication Type]) OR Comment[Publication Type]) OR Editorial[Publication Type]) OR Guideline[Publication Type]) OR Letter[Publication Type]) OR News[Publication Type]) OR Newspaper Article[Publication Type]) OR Review[Publication Type]	7,097,559
#13	#11 NOT #12	4,819
#14	Filters: Humans	4,484

© 2022 Ngamvichchukorn T et al. *JAMA Network Open.*

eTable 1. Systematic Review Search Strategy (Continued)

**Cochrane Library (From Inception to June 28, 2022)**		
**Search**	**Query**	**Items Found**
#1	MeSH descriptor: [Renal Dialysis] explode all trees	5,467
#2	MeSH descriptor: [Dialysis] explode all trees	235
#3	MeSH descriptor: [Peritoneal Dialysis] explode all trees	919
#4	MeSH descriptor: [Peritoneal Dialysis, Continuous Ambulatory] explode all trees	457
#5	intradialy* OR hemodialy* OR hemofilt* OR in-center dialysis OR nocturnal dialysis OR home dialysis OR peritoneal dialysis OR automated peritoneal dialysis OR continuous ambulatory peritoneal dialysis OR CAPD	15,755
#6	#1 OR #2 OR #3 OR #4 OR #5	16,963
#7	MeSH descriptor: [Kidney Transplantation] explode all trees	3,695
#8	kidney transplant* OR renal transplant* OR kidney recipient OR renal recipient	18,511
#9	#7 OR #8	18,511
#10	#6 AND #9	2,479
#11	graft function OR graft rejection OR graft survival OR graft failure OR acute rejection OR delayed rejection	16,055
#12	death OR mortality OR survival OR cardiovascular OR glomerular filtration OR hospitalization OR infection* OR re-transplant* OR vascular thrombosis OR costs OR health-related quality of life OR HRQOL OR QOL OR physical function OR psychological OR mental health OR psychosocial OR patient-reported outcomes	581,474
#13	#11 OR #12	585,343
#14	#10 AND #13	1,384
#15	Limit to Trials	1,228

© 2022 Ngamvichchukorn T et al. *JAMA Network Open.*

eTable 1. Systematic Review Search Strategy (Continued)

Scopus (From Inception to March 18, 2022)		
**Search**	**Query**	**Items Found**
#1	TITLE-ABS-KEY (“renal dialysis” OR dialysis OR hemodialysis OR “peritoneal dialysis” OR intradialy* OR hemodialy* OR hemofilt* OR “in-center dialysis” OR “nocturnal dialysis” OR “home dialysis” OR “automated peritoneal dialysis” OR “continuous ambulatory peritoneal dialysis” OR CAPD)	275,220
#2	TITLE-ABS-KEY (“renal transplant*” OR “kidney transplant” OR “kidney recipient” OR “renal recipient”)	78,984
#3	#1 AND #2	16,481
#4	TITLE-ABS-KEY (“graft function” OR “graft rejection” OR “graft survival” OR “graft failure” OR “acute rejection” OR “delayed rejection”)	182,872
#5	TITLE-ABS-KEY (death OR mortality OR survival OR cardiovascular OR glomerular filtration OR hospitalization OR infection* OR re-transplant* OR “vascular thrombosis” OR costs OR “health-related quality of life” OR HRQOL OR QOL OR “physical function” OR psychological OR “mental health” OR psychosocial OR “patient-reported outcomes”)	999,427
#6	#4 OR #5	1,150,606
#7	#3 AND #6	8,194
#8	(EXCLUDE (DOCTYPE , "re") OR EXCLUDE (DOCTYPE , "le") OR EXCLUDE (DOCTYPE , "sh") OR EXCLUDE (DOCTYPE , "ch") OR EXCLUDE (DOCTYPE , "ed") OR EXCLUDE (DOCTYPE , "no") OR EXCLUDE (DOCTYPE , "er") OR EXCLUDE (DOCTYPE , "bk")) AND (EXCLUDE (SRCTYPE , "k") OR EXCLUDE (SRCTYPE , "d"))	6,950

© 2022 Ngamvichchukorn T et al. *JAMA Network Open.*

eTable 1. Systematic Review Search Strategy (Continued)

**CINAHL (From Inception to March 18, 2022)**		
**Search**	**Query**	**Items Found**
#1	AB (“renal dialysis” OR dialysis OR hemodialysis OR “peritoneal dialysis” OR intradialy* OR hemodialy* OR hemofilt* OR “in-center dialysis” OR “nocturnal dialysis” OR “home dialysis” OR “automated peritoneal dialysis” OR “continuous ambulatory peritoneal dialysis” OR CAPD)	23,742
#2	AB (“renal transplant*” OR “kidney transplant” OR “kidney recipient” OR “renal recipient”)	5,930
#3	S1 AND S2	1,366
#4	AB (“graft function” OR “graft rejection” OR “graft survival” OR “graft failure” OR “acute rejection” OR “delayed rejection”)	4,341
#5	AB (death OR mortality OR survival OR cardiovascular OR glomerular filtration OR hospitalization OR infection* OR re-transplant* OR vascular thrombosis OR costs OR health-related quality of life OR HRQOL OR QOL OR physical function OR psychological OR mental health OR psychosocial OR patient-reported outcomes)	1,115,632
#6	S4 OR S5	1,117,091
#7	S3 AND S6	897
#8	Limiters<br>Source Types: Academic Journals<br>Narrow by Subject Age: - all child<br>Narrow by Subject Age: - all adult<br>Expanders - Apply equivalent subjects<br>Search modes - Boolean/Phrase	512

© 2022 Ngamvichchukorn T et al. *JAMA Network Open.*

eTable 2. The PICOTS Format: Study Inclusion/Exclusion Criteria

Elements	Criteria for Inclusion	Criteria for Exclusion
Populations	• Kidney transplant recipients regardless of age, donor sources (living or deceased donor), and comorbid conditions<br>• Other subgroups analysis was also included if the studies providing data to calculate the effect estimates of the outcome of interest	• In vitro or animal studies
Interventions	• Dialysis modalities: in-center HD (conventional, short daily, and nocturnal), home HD, home PD (APD and CAPD)	• Studies recruiting participants who received both PD and HD treatment
Comparators	• Any type of mode of pretransplant dialysis treatment	• Studies without control groups
Outcomes	• Primary outcomes<br>❖ All-cause mortality<br>❖ Overall graft failure<br>❖ Death-censored graft failure<br>❖ Delayed graft function<br>• Secondary outcomes<br>❖ Acute rejection<br>❖ Graft vessel thrombosis<br>❖ Oliguria (not producing urine in the first 24 hours)<br>❖ de novo heart failure<br>❖ NODAT<br>• Additional outcomes<br>❖ Changes in estimated glomerular filtration rate<br>❖ All-cause hospitalization<br>❖ Re-transplantation<br>❖ Re-entry of chronic dialysis<br>❖ HRQOL	• Studies not providing data to calculate the effect estimates of the outcome of interest
Timing	• An extensive search strategy from the inception of bibliographic databases forward to assure all published literature was identified	• No limit timing of start date
Setting	• Published RCTs, quasi-RCT, and comparative effectiveness observational studies (cohort studies and case-control studies) in any setting and context<br>• Gray literature, ongoing trial, and preprint data were browsed<br>• Studies will not be limited language	• Crossover, cross-sectional, N-of-one trial, case series/case reports, and phase I or II study design<br>• Reports not involving primary data including, narrative review, systematic review, meta-analysis, news items, consensus statement, guidelines, and opinion/editorials

© 2022 Ngamvichchukorn T et al. *JAMA Network Open.*

Abbreviations: APD, automated peritoneal dialysis; CAPD, continuous ambulatory peritoneal dialysis; HD, hemodialysis; HRQOL, health-related quality of life; NODAT, new onset diabetes mellitus after transplantation; PD, peritoneal dialysis; PICOTS, populations, interventions, comparators, outcomes, timing, setting; RCTs, randomized controlled trials.

© 2022 Ngamvichchukorn T et al. *JAMA Network Open.*

eTable 3. Characteristics of Study Participants Included in the Meta-Analysis

First Author (Year)	Race, No (%)	BMI at Transplant in kg/m^2, Mean (SD)	HLA Mismatch, No. (%)	PRA Titer, Mean (SD)	Cause of ESKD, No (%)	HTN, No. (%)	Diabetes, No. (%)	CAD, No. (%)	Cerebro-vascular Disease, No. (%)	PVD, No. (%)	Cancer, No. (%)
Pérez Fontán et al[1] (1998)	NR	NR	HLA-A mismatches: 1.3	NR	NR	NR	42 (5.1%)	NR	NR	NR	NR
Bleyer et al[2] (1999)	White: 80% in PD; 67% in HD	NR	0-2 (50% in PD, 55% in HD); 3-4 (36% in PD, 33% in HD); 5-6 (13% in PD, 11% in HD)	12.9 (23.5) in PD; 16.3 (26.9) in HD	NR	NR	NR	NR	NR	NR	NR
Ojo et al[3] (1999)[†]	White, 1643 (73.9%); Black, 475 (21.4%); Other race, 105 (4.7%)	NR	NR	16.9 (27.5)	Diabetes, 505 (22.7%); HTN, 308 (13.8%); GN, 616 (27.7%); Other, 794 (35.7%)	NR	505 (22.7%)	NR	NR	NR	NR
Van Biesen et al[4] (2000)	NR	NR	NR	NR	NR	NR	NR	NR	NR	NR	NR
Snyder et al[5] (2002)	White, 12746 (56.0%); Black, 6670 (29.3%); Other race, 3360 (14.7%)	>29, 3431 (15.1%)	NR	NR	Diabetes, 10077 (44.2%)	14748 (64.8%)	10077 (44.2%)	8833 (38.8%)	NR	2848 (12.5%)	NR
Chalem et al[6] (2005)	NR	NR	3.1 (1.2)	0-14 (n=2928, 93.3%); 15-69 (n=159, 5.1%); ≥70 (n=51, 1.6%)	NR	NR	110 (3.5%)	NR	NR	NR	NR
Fontana et al[7] (2005)	NR	19.4 (5.5)	NR	NR	ICRF, 61 (37.2%); IARF, 20 (12.2%); hereditary, 32 (19.5%); congenital	NR	NR	NR	NR	NR	NR

© 2022 Ngamvichchukorn T et al. *JAMA Network Open.*

					(11.0%); Other, 33 (20.1%)						

†On the basis of the whole sample (743 cases and 1,480 controls).

Abbreviations: BMI, body mass index; CAD, coronary artery disease; ESKD, end-stage kidney disease; GN, glomerulonephritis; HD, hemodialysis; HLA, human leukocyte antigens; HTN, hypertension; IARF, irreversible acute renal failure; ICRF, irreversible chronic renal failure; NR, not reported; PD, peritoneal dialysis; PRA, panel reactive antibody; PVD, peripheral vascular disease; SD, standard deviation.

© 2022 Ngamvichchukorn T et al. *JAMA Network Open.*

eTable 3. Characteristics of Study Participants Included in the Meta-Analysis (Continued)

First Author (Year)	Race, No (%)	BMI at Transplant in kg/m^2, Mean (SD)	HLA Mismatch, No. (%)	PRA Titer, Mean (SD)	Cause of ESKD, No (%)	HTN, No. (%)	Diabetes, No. (%)	CAD, No. (%)	Cerebro-vascular Disease, No. (%)	PVD, No. (%)	Cancer, No. (%)
Goldfarb-Rumyantzev et al[8] (2005)‡	White, 65176 (70.2%); Black, 21354 (23.0%); Asian, 3157 (3.4%); Other, 3157 (3.4%)	25.4 (9.2)	1.8 (1.5)	12.1 (21.5)	Diabetes, 23397 (25.2%); HTN, 15969 (17.2%); GN, 23954 (25.8%); Other, 29524 (31.8%)	48743 (52.5%)	25254 (27.2%)	NR	NR	NR	NR
Resende et al[9] (2009)	NR	NR	0, (n=29, 6.9%); 1-6 (n=392, 93.1%)	<50% (n=380, 90.3%); ≥50% (n=41, 9.7%)	Diabetes, 28 (6.7%); HTN, 42 (10.0%); GN, 129 (30.6%); Other, 222 (52.7%)	NR	NR	NR	NR	NR	NR
Courivaud et al[10] (2011)	White, 1896 (100%)	23.1 (4.0)	NR	NR	NR	NR	0 (0.0%)	NR	NR	NR	NR
Madziarska et al[11] (2011)	White, 308 (100%)	23.8 (3.8)	3.5 (0.8)	NR	HTN, 52 (16.9%); GN, 154 (50.0%); Interstitial nephropathy, 42 (13.6%); PKD, 49 (15.9%); Other, 29524 (31.8%)	NR	0 (0.0%)	NR	NR	NR	NR
Schwenger et al[12] (2011)	White, 52812 (92.1%)	NR	0-1 (n=8247, 14.4%); 2-4 (n=39880, 69.6%); 5-6 (n=9188, 16.0%)	NR	NR	NR	5105 (8.9%)	NR	NR	NR	NR
Sezer et al[13] (2011)	NR	24.0 (10.5)	NR	NR	HTN, 66 (26.4%); GN, 59 (23.6%); Vesicoureteral reflux, 45 (18.0%); Other, 80 32.0%)	NR	NR	NR	NR	NR	NR

‡On the basis of the whole cohort (n=92,844).

© 2022 Ngamvichchukorn T et al. *JAMA Network Open.*

Abbreviations: BMI, body mass index; CAD, coronary artery disease; ESKD, end-stage kidney disease; GN, glomerulonephritis; HLA, human leukocyte antigens; HTN, hypertension; NR, not reported; PKD, polycystic kidney disease; PRA, panel reactive antibody; PVD, peripheral vascular disease; SD, standard deviation.

© 2022 Ngamvichchukorn T et al. *JAMA Network Open.*

eTable 3. Characteristics of Study Participants Included in the Meta-Analysis (Continued)

First Author (Year)	Race, No (%)	BMI at Transplant in kg/m^2, Mean (SD)	HLA Mismatch, No. (%)	PRA Titer, Mean (SD)	Cause of ESKD, No (%)	HTN, No. (%)	Diabetes, No. (%)	CAD, No. (%)	Cerebro-vascular Disease, No. (%)	PVD, No. (%)	Cancer, No. (%)
Kramer et al[14] (2012)	NR	NR	NR	NR	DM, 3985 (13.7%); HTN, 3054 (10.5%); GN, 7185 (24.7%); Other, 14864 (51.1%)	NR	NR	NR	NR	NR	NR
Molnar et al[15] (2012)	Black, 3677 (25.3%)	26.5 (6.0)	3.6 (1.8)	10.0 (24.0)	NR	NR	5026 (34.6%)	NR	NR	NR	NR
Lopez-Oliva et al[16] (2014)	NR	24.6 (4.1)	0 (n=83, 35.2%); 1 (n=135, 57.2%); 2 (n=20, 8.5%)	NR	Diabetes, 15 (6.3%); HTN, 16 (6.8%); GN, 76 (32.2%); PKD, 29 (12.3%); Other, 100 (42.4%)	NR	NR	NR	NR	NR	NR
Martins et al[17] (2015)	NR	22.4 (2.8)	4.5 (1.1)	NR	NR	NR	158 (100.0%)	29 (18.4%)	NR	NR	NR
Dipalma et al[18] (2016)	NR	25.1 (5.5)	3.3 (0.8)	>10% (n=29, 18.1%)	Diabetes, 16 (10.0%); HTN, 31 (19.4%); GN, 32 (20.0%); PKD, 18 (11.2%); Other, 63 (39.4%)	111 (69.4%)	25 (15.6%)	16 (10.0%)	6 (3.8%)	6 (3.8%)	6 (3.8%)
Dębska-Ślizień et al[19] (2018)	NR	NR	3.0 (NS)	NR	Diabetes, 40 (15.5); HTN, 35 (13.2%); GN, 93 (35.0%); Chronic interstitial nephritis, 18 (6.8%), PKD, 25 (9.4%); Other, 55 (20.7%)	NR	NR	NR	NR	NR	NR

Abbreviations: BMI, body mass index; CAD, coronary artery disease; ESKD, end-stage kidney disease; GN, glomerulonephritis; HLA, human leukocyte antigens; HTN, hypertension; NR, not reported; NS, not specified; PKD, polycystic kidney disease; PRA, panel reactive antibody; PVD, peripheral vascular disease; SD, standard deviation.

© 2022 Ngamvichchukorn T et al. *JAMA Network Open.*

eTable 3. Characteristics of Study Participants Included in the Meta-Analysis (Continued)

First Author (Year)	Race, No (%)	BMI at Transplant in kg/m^2, Mean (SD)	HLA Mismatch, No. (%)	PRA Titer, Mean (SD)	Cause of ESKD, No (%)	HTN, No. (%)	Diabetes, No. (%)	CAD, No. (%)	Cerebro-vascular Disease, No. (%)	PVD, No. (%)	Cancer, No. (%)
Lin et al[20] (2018)	Asian, 1812 (100.0%)	NR	NR	NR	NR	1416 (78.1%)	274 (15.1%)	280 (15.4%)	94 (5.2%)	NR	49 (2.7%)
Marcacuzco et al[21] (2018)	NR	23.7 (3.7)	NR	NR	NR	NR	165 (100.0%)	NR	NR	NR	NR
Balzer et al[22] (2020)	NR	24.8 (3.9)	NR	9.7 (26.1)	Diabetes, 272 (13.6%); HTN, 123 (6.1%); GN, 526 (26.2%); Other, 1085 (54.1%)	NR	NR	468 (23.3%)	NR	NR	NR
Scheuermann et al[23] (2020)	NR	24.9 (4.2)	NR	NR	NR	NR	83 (100.0%)	23 (27.7%)	NR	NR	NR
Lenihan et al[24] (2021)	White, 16313 (58.9%); Black, 8909 (32.2%); Others, 2440 (8.8%)	27.9 (5.2)	0 (n=2099, 7.6%); 1-3 (n=6413, 23.2%); 4-6 (n=18668, 67.4%)	14.7 (26.8)	Diabetes, 6991 (25.2%); HTN, 7187 (25.9%); GN, 6905 (24.9%); Other, 6519 (23.5%)	25536 (92.2%)	11229 (40.5%)	6835 (24.7%)	1903 (6.9%)	5233 (18.9%)	1839 (6.6%)
So et al[25] (2021)	White, 674 (84.0%); Asian, 76 (9.5%); Other/unknown, 52 (6.5%)	27.5 (4.7)	NR	NR	Diabetes, 147 (18.3%); HTN, 107 (13.3%); GN, 276 (34.4%); Other/unknown, 272 (34.0%)	NR	299 (37.3%)	302 (37.7%)	91 (11.4%)	153 (19.1%)	NR
Prezelin-Reydit et al[26] (2021)[§]	NR	NR	0-2 (n=317, 21.6%); 3-4 (n=977, 66.7%); 5-6 (n=171, 11.7%)	NR	Other/unknown, 248 (16.2%); GN, 468 (30.6%); Hereditary, 265 (17.3%); Vascular, 100 (6.5%); Congenital, 450 (29.4%)	NR	NR	NR	NR	NR	NR

[§]On the basis of non-preemptive kidney transplantation cohort.

© 2022 Ngamvichchukorn T et al. *JAMA Network Open.*

Abbreviations: BMI, body mass index; CAD, coronary artery disease; ESKD, end-stage kidney disease; GN, glomerulonephritis; HLA, human leukocyte antigens; HTN, hypertension; NR, not reported; PRA, panel reactive antibody; PVD, peripheral vascular disease; SD, standard deviation.

© 2022 Ngamvichchukorn T et al. *JAMA Network Open.*

eTable 4. Risk of Bias Assessment of Included Studies by the NOS

**Cohort Studies**										
**First Author, Year**	**Selection**				**Comparability**		**Outcomes**			**Total NOS**
	**Representativeness**	**Non-Exposed: Selection**	**Exposure: Ascertainment**	**Outcomes Not Present at Entry**	**Controls for: Donor age, type of donor, CIT**	**Control for: additional Factors†**	**Assessment**	**Follow-up Long Enough**	**Adequacy of follow-up**	
Pérez Fontán et al[1] (1998)	*	*	…	*	*	…	*	*	*	7
Bleyer et al[2] (1999)	*	*	…	*	*	*	*	*	*	8
Van Biesen et al[4] (2000)	*	*	*	*	…	…	*	*	*	7
Snyder et al[5] (2002)	*	*	*	*	*	*	*	*	*	9
Chalem et al[6] (2005)	*	*	…	*	*	*	*	*	*	8
Fontana et al[7] (2005)	*	*	*	*	…	…	*	*	*	7
Goldfarb-Rumyantzev et al[8] (2005)	*	*	*	*	…	*	*	*	*	8
Resende et al[9] (2009)	*	*	…	*	…	…	*	*	*	6
Courivaud et al[10] (2011)	*	*	…	*	…	…	*	*	*	6
Madziarska et al[11] (2011)	*	*	…	*	…	…	*	*	*	6
Schwenger et al[12] (2011)	*	*	…	*	*	*	*	*	*	8
Sezer et al[13] (2011)	*	*	*	*	…	…	*	*	*	6
Kramer et al[14] (2012)	*	*	*	*	…	…	*	*	*	7
Molnar et al[15] (2012)	*	*	*	*	*	*	*	*	*	9
Lopez-Oliva et al[16] (2014)	*	*	…	*	…	…	*	*	*	6
Martins et al[17] (2015)	*	*	…	*	…	*	*	*	*	7

†Study control for 3 of additional factors: panel reactive antibody, HLA mismatch, dialysis duration, co-morbidity
Abbreviations: CIT, cold ischemic time; HLA, human leukocyte antigens; NOS, Newcastle-Ottawa Scale.

© 2022 Ngamvichchukorn T et al. *JAMA Network Open.*

eTable 4. Risk of Bias Assessment of Included Studies by the NOS (Continued)

**Cohort Studies (Continued)**										
**First Author, Year**	**Selection**				**Comparability**		**Outcomes**			**Total NOS**
	**Representativeness**	**Non-Exposed: Selection**	**Exposure: Ascertainment**	**Outcomes Not Present at Entry**	**Controls for: Donor age, type of donor, CIT**	**Control for: additional Factors†**	**Assessment**	**Follow-up Long Enough**	**Adequacy of follow-up**	
Dipalma et al[18] (2016)	*	*	…	*	…	*	*	*	*	7
Dębska-Ślizień et al[19] (2018)	*	*	*	*	…	…	…	*	*	6
Lin et al[20] (2018)	*	*	*	*	…	…	*	*	*	7
Marcacuzco et al[21] (2018)	*	*	…	*	…	…	*	*	*	6
Balzer et al[22] (2020)	*	*	*	*	*	*	*	*	*	9
Scheuermann et al[23] (2020)	*	*	…	*	…	*	*	*	*	7
Lenihan et al[24] (2021)	*	*	*	*	*	*	*	*	*	9
So et al[25] (2021)	*	*	…	*	…	…	*	*	*	6
Prezelin-Reydit et al[26] (2021)	*	*	…	*	*	…	*	*	*	7
**Case-Control Studies**										
**First Author, Year**	**Selection**				**Comparability**		**Exposure**			**Total NOS**
	**Cases: Definition**	**Cases: Representativeness**	**Controls: Selection**	**Controls: Definitions**	**Controls for: Donor age, type of donor, CIT**	**Control for: additional Factors†**	**Ascertainment**	**Same Method**	**Non-Response Rate**	
Ojo et al[3] (1999)	*	*	*	*	…	*	*	*	*	8

†Study control for 3 of additional factors: panel reactive antibody, HLA mismatch, dialysis duration, co-morbidity

Abbreviations: CIT, cold ischemic time; HLA, human leukocyte antigens; NOS, Newcastle-Ottawa Scale.

© 2022 Ngamvichchukorn T et al. *JAMA Network Open.*

eTable 5. Subgroup Analysis of Primary Outcomes

Subgroup Comparison: All-Cause Mortality (PD vs. HD)	No. of Studies (Ref)	No. of Participants	HR (95% CI)	*P* Value	Heterogeneity			
					Q Statistic	*P* Value	I^2 Index (95% CI)	τ^2
**Study Population**								
Adult	12 (5, 12, 14, 15, 16, 17, 18, 20, 21, 22, 23, 25)	128,971	0.92 (0.81 – 1.06)	0.261	36.04	<0.001	69.5% (36.1 – 81.7)	0.022
Pediatric/mixed cases	1 (8)	92,844	0.94 (0.91 – 0.97)	<0.001	NA	NA	NA	NA
**Donor Type**								
Deceased	4 (12, 17, 18, 23)	57,578	1.34 (0.62 – 2.91)	0.463	7.57	0.056	60.4% (0.0 – 84.6)	0.357
Mixed cases/unspecified	9 (5, 8, 14, 15, 16, 20, 21, 22, 25)	164,237	0.92 (0.81 – 1.03)	0.144	30.64	<0.001	73.9 (39.8 – 85.0)	0.014
**Publication Date**								
Before 2015	6 (5, 8, 12, 14, 15, 17)	216,767	0.89 (0.83 – 0.96)	0.002	15.52	0.008	67.8% (0.0 – 84.4)	0.004
2015 to 2022	7 (17, 18, 20, 21, 22, 23, 25)	5,048	1.24 (0.81 – 1.89)	0.322	17.80	0.007	66.3% (0.0 – 83.0)	0.172
**Sample size**								
≤1,000	6 (16, 17, 18, 21, 23, 25)	1,466	1.27 (0.67 – 2.42)	0.460	13.67	0.018	63.4% (0.0 – 82.9)	0.371
>1,000	7 (5, 8, 12, 14, 15, 20, 22)	220,349	0.90 (0.84 – 0.96)	0.003	17.15	0.009	65.0% (0.0 – 82.5)	0.004
**Study Setting**								
Single-center	6 (16, 17, 18, 21, 22, 23)	2,670	0.98 (0.52 – 1.86)	0.960	13.05	0.023	61.7% (0.0 – 82.2)	0.356
Multicenter	7 (5, 8, 12, 14, 15, 20, 25)	219,145	0.93 (0.85 – 1.01)	0.086	24.14	<0.001	75.1% (33.5 – 86.6)	0.007
**Study Location**								
European	6 (16, 17, 18, 21, 22, 23)	2,670	0.98 (0.52 – 1.86)	0.960	13.05	0.023	61.7% (0.0 – 82.2)	0.356

© 2022 Ngamvichchukorn T et al. *JAMA Network Open.*

Non-European region/international	7 (5, 8, 12, 14, 15, 20, 25)	219,145	0.93 (0.85 – 1.01)	0.086	24.14	<0.001	75.1% (33.5 – 86.6)	0.007

Abbreviations: CI, confidence interval; HD, hemodialysis; HR, hazard ratio; NA, not applicable; PD, peritoneal dialysis.

© 2022 Ngamvichchukorn T et al. *JAMA Network Open.*

eTable 5. Subgroup Analysis of Primary Outcomes (Continued)

Subgroup Comparison: Overall Graft Failure (PD vs. HD)	No. of Studies (Ref)	No. of Participants	HR (95% CI)	*P* Value	Heterogeneity			
					Q Statistic	*P* Value	I^2 Index (95% CI)	τ^2
**Study Population**								
Adult	8 (5, 6, 9, 12, 14, 16, 22, 23)	117,762	0.96 (0.90 – 1.03)	0.243	13.51	0.061	48.2% (0.0 – 75.2)	0.003
Pediatric/mixed cases	2 (8, 26)	94,224	0.97 (0.94 – 0.99)	0.018	0.30	0.584	0.0% (NA)	<0.001
**Donor Type**								
Deceased	4 (6, 9, 12, 23)	63,656	0.94 (0.90 – 0.98)	0.010	0.17	0.983	0.0% (0.0 – 67.9)	<0.001
Mixed cases/unspecified	6 (5, 8, 14, 16, 22, 26)	148,330	0.97 (0.91 – 1.02)	0.212	13.18	0.022	62.1% (0.0 – 82.4)	0.002
**Publication Date**								
Before 2015	7 (5, 6, 8, 9, 12, 14, 16)	208,517	0.96 (0.92 – 1.01)	0.120	13.04	0.042	54.0% (0.0 – 78.4)	0.002
2015 to 2022	3 (22, 23, 26)	3,469	0.94 (0.88 – 1.01)	0.096	1.05	0.590	0.0% (0.0 – 72.9)	<0.001
**Sample size**								
≤1,000	3 (9, 16, 23)	740	1.24 (0.86 – 1.77)	0.244	0.96	0.618	0.0% (0.0 – 72.9)	<0.001
>1,000	7 (5, 6, 8, 12, 14, 22, 26)	211,246	0.96 (0.92 – 0.99)	0.018	11.45	0.076	47.6% (0.0 – 76.1)	0.001
**Study Setting**								
Single-center	4 (9, 16, 22, 23)	2,746	1.02 (0.74 – 1.42)	0.897	3.93	0.269	23.7% (0.0 – 74.8)	0.027
Multicenter	6 (5, 6, 8, 12, 14, 26)	209,240	0.96 (0.92 – 0.99)	0.022	10.28	0.068	51.4% (0.0 – 78.7)	0.001
**Study Location**								
European	6 (6, 9, 16, 22, 23, 26)	9,963	0.95 (0.89 – 1.02)	0.133	4.20	0.521	0.0% (0.0 – 61.0)	<0.001
Non-European region/international	4 (5, 8, 12, 14)	202,023	0.96 (0.92 – 1.01)	0.100	10.05	0.018	70.2% (0.0 – 87.5)	0.002

© 2022 Ngamvichchukorn T et al. *JAMA Network Open.*

Abbreviations: CI, confidence interval; HD, hemodialysis; HR, hazard ratio; NA, not applicable; PD, peritoneal dialysis.

© 2022 Ngamvichchukorn T et al. *JAMA Network Open.*

eTable 5. Subgroup Analysis of Primary Outcomes (Continued)

Subgroup Comparison: Death-Censored Graft Failure (PD vs. HD)	No. of Studies (Ref)	No. of Participants	HR (95% CI)	*P* Value	Heterogeneity			
					Q Statistic	*P* Value	I^2 Index (95% CI)	τ^2
**Study Population**								
Adult	5 (5, 12, 15, 18, 20)	96,439	0.98 (0.85 – 1.14)	0.811	15.23	0.004	73.7% (0.0 – 87.5)	0.016
Pediatric/mixed cases	NA	NA	NA	NA	NA	NA	NA	NA
**Donor Type**								
Deceased	2 (12, 18)	57,343	0.93 (0.72 – 1.20)	0.598	1.18	0.278	14.9% (NA)	0.017
Mixed cases/unspecified	3 (5, 15, 20)	39,096	0.98 (0.75 – 1.30)	0.915	8.74	0.013	77.1% (0.0 – 90.9)	0.045
**Publication Date**								
Before 2015	3 (5, 12, 15)	94,599	1.06 (0.92 – 1.21)	0.453	8.66	0.013	76.9% (0.0 – 90.9)	0.010
2015 to 2022	2 (18, 20)	1,840	0.71 (0.54 – 0.94)	0.015	0.16	0.685	0.0% (NA)	<0.001
**Sample size**								
≤1,000	1 (18)	28	0.60 (0.25 – 1.43)	0.248	NA	NA	NA	NA
>1,000	4 (5, 12, 15, 20)	96,411	1.00 (0.86 – 1.16)	0.960	13.84	0.003	78.3% (2.7 – 90.0)	0.015
**Study Setting**								
Single-center	1 (18)	28	0.60 (0.25 – 1.43)	0.248	NA	NA	NA	NA
Multicenter	4 (5, 12, 15, 20)	96,411	1.00 (0.86 – 1.16)	0.960	13.84	0.003	78.3% (2.7 – 90.0)	0.015
**Study Location**								
European	1 (18)	28	0.60 (0.25 – 1.43)	0.248	NA	NA	NA	NA
Non-European region/international	4 (5, 12, 15, 20)	96,411	1.00 (0.86 – 1.16)	0.960	13.84	0.003	78.3% (2.7 – 90.0)	0.015

Abbreviations: CI, confidence interval; HD, hemodialysis; HR, hazard ratio; NA, not applicable; PD, peritoneal dialysis.

© 2022 Ngamvichchukorn T et al. *JAMA Network Open.*

eTable 5. Subgroup Analysis of Primary Outcomes (Continued)

Subgroup Comparison: Delayed Graft Function (PD vs. HD)	No. of Studies (Ref)	No. of Participants	OR (95% CI)	*P* Value	Heterogeneity			
					Q Statistic	*P* Value	I^2 Index (95% CI)	τ^2
**Study Population**								
Adult	5 (2, 4, 5, 13, 15)	46,944	0.73 (0.69 – 0.77)	<0.001	5.35	0.254	25.2% (0.0 – 72.4)	<0.001
Pediatric/mixed cases	1 (7)	174	0.83 (0.47 – 1.49)	0.536	0.00	NA	NA	NA
**Donor Type**								
Deceased	3 (2, 4, 7)	9,584	0.71 (0.63 – 0.79)	<0.001	0.89	0.639	0.0% (0.0 – 72.9)	<0.001
Mixed cases/unspecified	3 (5, 13, 15)	37,534	0.74 (0.69 – 0.81)	<0.001	4.53	0.104	55.8% (0.0 – 85.8%)	0.003
**Publication Date**								
Before 2015	6 (2, 4, 5, 7, 13, 15)	47,118	0.73 (0.70 – 0.76)	<0.001	5.58	0.349	10.4% (0.0 – 64.9)	<0.001
2015 to 2022	NA	NA	NA	NA	NA	NA	NA	NA
**Sample size**								
≤1,000	3 (4, 7, 13)	543	0.72 (0.70 – 0.74)	<0.001	0.95	0.621	0.0% (0.0 – 72.9)	<0.001
1,000	3 (2, 5, 15)	46,575	0.76 (0.68 – 0.84)	<0.001	3.79	0.150	47.2% (0.0 – 83.9)	0.004
**Study Setting**								
Single-center	3 (4, 7, 13)	543	0.72 (0.70 – 0.74)	<0.001	0.95	0.621	0.0% (0.0 – 72.9)	<0.001
Multicenter	3 (2, 5, 15)	46,575	0.76 (0.68 – 0.84)	<0.001	3.79	0.150	47.2% (0.0 – 83.9)	0.004
**Study Location**								
European	3 (4, 7, 13)	543	0.72 (0.70 – 0.74)	<0.001	0.95	0.621	0.0% (0.0 – 72.9)	<0.001
Non-European region/international	3 (2, 5, 15)	46,575	0.76 (0.68 – 0.84)	<0.001	3.79	0.150	47.2% (0.0 – 83.9)	0.004

Abbreviations: CI, confidence interval; HD, hemodialysis; NA, not applicable; OR, odds ratio; PD, peritoneal dialysis.

© 2022 Ngamvichchukorn T et al. *JAMA Network Open.*

eTable 6. Sensitivity Analysis: Restricting the Analysis to Studies That Adjusted for Key Confounding Factors[†]

Kidney Transplantation Outcomes (PD vs. HD)	No. of Studies (Ref)	No. of Participants	Effect Estimate (95% CI)	*P* Value	Heterogeneity			
					Q Statistic	*P* Value	I^2 Index (95% CI)	τ^2
**Primary Outcomes**								
All-cause mortality	4 (5, 12, 15, 22)	96,605	HR 0.86 (0.75 – 0.99)	0.031	8.12	0.044	63.1% (0.0 – 85.4)	0.010
Overall graft failure	5 (5, 6, 12, 22, 26)	89,314	HR 0.97 (0.91 – 1.02)	0.259	7.41	0.116	46.0% (0.0 – 78.6)	0.002
Death-censored graft failure	3 (5, 12, 15)	94,599	HR 1.06 (0.92 – 1.21)	0.453	8.66	0.013	76.9% (0.0 – 90.9)	0.010
Delayed graft function	3 (2, 5, 15)	46,575	OR 0.76 (0.68 – 0.84)	<0.001	3.79	0.150	47.2% (0.0 – 83.9)	0.004
**Secondary Outcomes**								
Acute rejection	1 (22)	2,006	OR 0.70 (0.51 – 0.96)	0.029	NA	NA	NA	NA
Graft vessel thrombosis	1 (1)	827	OR 0.40 (0.13 – 1.24)	0.113	NA	NA	NA	NA
Oliguria (not producing urine in the first 24 hours)	1 (2)	9,291	OR 0.74 (0.62 – 0.87)	<0.001	NA	NA	NA	NA
de novo heart failure	1 (24)	27,701	HR 0.84 (0.78 – 0.91)	<0.001	NA	NA	NA	NA
NODAT	NA	NA	NA	NA	NA	NA	NA	NA

[†]To include donor age, type of donor, cold ischemic time.

Abbreviations: CI, confidence interval; HD, hemodialysis; HR, hazard ratio; NA, not applicable; NODAT, new onset diabetes mellitus after transplantation; OR, odds ratio; PD, peritoneal dialysis.

© 2022 Ngamvichchukorn T et al. *JAMA Network Open.*

eTable 7. Sensitivity Analysis: Restricting the Analysis to Studies Judged to Be of the Highest Quality (NOS ≥8 Points)

Kidney Transplantation Outcomes (PD vs. HD)	No. of Studies (Ref)	No. of Participants	Effect Estimate (95% CI)	*P* Value	Heterogeneity			
					Q Statistic	*P* Value	I^2 Index (95% CI)	τ^2
**Primary Outcomes**								
All-cause mortality	5 (5, 8, 12, 15, 22)	189,449	HR 0.91 (0.85 – 0.98)	0.009	9.27	0.055	56.8% (0.0 – 82.0)	0.003
Overall graft failure	5 (5, 6, 8, 12, 22)	180,778	HR 0.97 (0.93 – 1.02)	0.216	7.24	0.124	44.8% (0.0 – 78.3)	0.001
Death-censored graft failure	3 (5, 12, 15)	94,599	HR 1.06 (0.92 – 1.21)	0.453	8.66	0.013	76.9% (0.0 – 90.9)	0.010
Delayed graft function	3 (2, 5, 15)	46,575	OR 0.76 (0.68 – 0.84)	<0.001	3.79	0.150	47.2% (0.0 – 83.9)	0.004
**Secondary Outcomes**								
Acute rejection	1 (22)	2,006	OR 0.70 (0.51 – 0.96)	0.029	NA	NA	NA	NA
Graft vessel thrombosis	1 (3)	1,991	OR 1.87 (1.28 – 2.73)	0.001	NA	NA	NA	NA
Oliguria (not producing urine in the first 24 hours)	1 (2)	9,291	OR 0.74 (0.62 – 0.87)	<0.001	NA	NA	NA	NA
de novo heart failure	1 (24)	27,701	HR 0.84 (0.78 – 0.91)	<0.001	NA	NA	NA	NA
NODAT	NA	NA	NA	NA	NA	NA	NA	NA

Abbreviations: CI, confidence interval; HD, hemodialysis; HR, hazard ratio; NA, not applicable; NODAT, new onset diabetes mellitus after transplantation; OR, odds ratio; PD, peritoneal dialysis.

© 2022 Ngamvichchukorn T et al. *JAMA Network Open.*

eTable 8. Sensitivity Analysis: Including the Analysis of Studies With the Directness of Effect Estimates

Kidney Transplantation Outcomes (PD vs. HD)	No. of Studies (Ref)	No. of Participants	Effect Estimate (95% CI)	*P* Value	Heterogeneity			
					Q Statistic	*P* Value	I^2 Index (95% CI)	τ^2
**Primary Outcomes**								
All-cause mortality	12 (5, 8, 12, 14, 15, 16, 17, 18, 20, 21, 22, 23)	221,013	HR 0.90 (0.83 – 0.97)	0.010	28.44	0.003	61.3% (11.7 – 77.9)	0.007
Overall graft failure	8 (5, 6, 8, 12, 14, 16, 22, 23)	210,185	HR 0.96 (0.92 – 1.01)	0.093	14.17	0.048	50.6% (0.0 – 76.1)	0.002
Death-censored graft failure	5 (5, 12, 15, 18, 20)	96,439	HR 0.98 (0.85 – 1.14)	0.811	15.23	0.004	73.7% (0.0 – 87.5)	0.016
Delayed graft function	6 (2, 4, 5, 7, 13, 15)	47,118	OR 0.73 (0.70 – 0.76)	<0.001	5.58	0.349	10.4% (0.0 – 64.9)	<0.001
**Secondary Outcomes**								
Acute rejection	1 (22)	2,006	OR 0.70 (0.51 – 0.96)	0.029	NA	NA	NA	NA
Graft vessel thrombosis	2 (1, 19)	1,093	OR 1.07 (0.15 – 7.57)	0.947	5.44	0.020	81.6% (NA)	1.629
Oliguria (not producing urine in the first 24 hours)	1 (2)	9,291	OR 0.74 (0.62 – 0.87)	<0.001	NA	NA	NA	NA
de novo heart failure	1 (24)	27,701	HR 0.84 (0.78 – 0.91)	<0.001	NA	NA	NA	NA
NODAT	2 (10, 11)	2,204	Or 1.57 (0.56 – 4.45)	0.393	5.48	0.019	81.8% (NA)	0.463

Abbreviations: CI, confidence interval; HD, hemodialysis; HR, hazard ratio; NA, not applicable; NODAT, new onset diabetes mellitus after transplantation; OR, odds ratio; PD, peritoneal dialysis.

© 2022 Ngamvichchukorn T et al. *JAMA Network Open.*

eTable 9. Sensitivity Analysis: Excluding Studies That Were Conducted Among SPKT Patients

**Kidney Transplantation Outcomes (PD vs. HD)**	**No. of Studies (Ref)**	**No. of Participants**	**Effect Estimate (95% CI)**	***P* Value**	**Heterogeneity**			
					***Q* Statistic**	***P* Value**	**I^2 Index (95% CI)**	**τ^2**
**Primary Outcomes**								
All-cause mortality	9 (5, 8, 12, 14, 15, 16, 18, 20, 25)	219, 403	HR 0.92 (0.84 – 1.01)	0.064	27.67	0.001	71.1% (30.2 – 83.8)	0.008
Overall graft failure	8 (5, 6, 8, 9, 12, 14, 16, 26)	209,897	HR 0.96 (0.92 – 0.99)	0.039	13.15	0.068	46.8% (0.0 – 74.6)	0.001
Death-censored graft failure	5 (5, 12, 15, 18, 20)	96,439	HR 0.98 (0.85 – 1.14)	0.811	15.23	0.004	73.7% (0.0 – 87.5)	0.016
Delayed graft function	6 (2, 4, 5, 7, 13, 15)	47,118	OR 0.73 (0.70 – 0.76)	<0.001	5.58	0.349	10.4% (0.0 – 64.9)	<0.001
**Secondary Outcomes**								
Acute rejection	NA	NA	NA	NA	NA	NA	NA	NA
Graft vessel thrombosis	3 (1, 3, 19)	3,084	OR 1.35 (0.50 – 3.65)	0.554	7.28	0.026	72.5% (0.0 – 89.7)	0.550
Oliguria (not producing urine in the first 24 hours)	1 (2)	9,291	OR 0.74 (0.62 – 0.87)	<0.001	NA	NA	NA	NA
de novo heart failure	1 (24)	27,701	HR 0.84 (0.78 – 0.91)	<0.001	NA	NA	NA	NA
NODAT	2 (10, 11)	2,204	Or 1.57 (0.56 – 4.45)	0.393	5.48	0.019	81.8% (NA)	0.463

Abbreviations: CI, confidence interval; HD, hemodialysis; HR, hazard ratio; NA, not applicable; NODAT, new onset diabetes mellitus after transplantation; OR, odds ratio; PD, peritoneal dialysis; SPKT, simultaneous pancreas-kidney transplantation.

© 2022 Ngamvichchukorn T et al. *JAMA Network Open.*

eTable 10. Sensitivity Analysis: Post-Hoc Analysis Using the "Leave-One-Out" Approach

First Author (Year)	HR (95% CI)			OR (95% CI)
	All-Cause Mortality	Overall Graft Failure	Death-Censored Graft Failure	Delayed Graft Function
**All studies**	**0.92 (0.84-1.01)**	**0.96 (0.92-0.99)**	**0.98 (0.85-1.14)**	**0.73 (0.70-0.76)**
Pérez Fontán et al[1] (1998)	NA	NA	NA	NA
Bleyer et al[2] (1999)	NA	NA	NA	0.74 (0.69-0.78)
Ojo et al[3] (1999)	NA	NA	NA	NA
Van Biesen et al[4] (2000)	NA	NA	NA	0.73 (0.70-0.76)
Snyder et al[5] (2002)	0.92 (0.82-1.02)	0.95 (0.92-0.98)	0.91 (0.76-1.09)	0.73 (0.68-0.78)
Chalem et al[6] (2005)	NA	0.96 (0.92-0.99)	NA	NA
Fontana et al[7] (2005)	NA	NA	NA	0.73 (0.69-0.77)
Goldfarb-Rumyantzev et al[8] (2005)	0.92 (0.81-1.06)	**0.96 (0.91-1.01)**	NA	NA
Resende et al[9] (2009)	NA	0.96 (0.92-0.99)	NA	NA
Courivaud et al[10] (2011)	NA	NA	NA	NA
Madziarska et al[11] (2011)	NA	NA	NA	NA
Schwenger et al[12] (2011)	0.93 (0.82-1.05)	**0.96 (0.92-1.01)**	0.95 (0.72-1.24)	NA
Sezer et al[13] (2011)	NA	NA	NA	0.74 (0.68-0.81)
Kramer et al[14] (2012)	0.94 (0.85-1.05)	**0.97 (0.94-1.00)**	NA	NA
Molnar et al[15] (2012)	0.94 (0.86-1.02)	NA	0.96 (0.81-1.14)	0.72 (0.70-0.74)
Lopez-Oliva et al[16] (2014)	0.93 (0.85-1.02)	0.96 (0.93-0.99)	NA	NA
Martins et al[17] (2015)	**0.91 (0.84-0.99)**	NA	NA	NA
Dipalma et al[18] (2016)	0.92 (0.84-1.01)	NA	1.00 (0.86-1.16)	NA
Dębska-Ślizień et al[19] (2018)	NA	NA	NA	NA
Lin et al[20] (2018)	**0.91 (0.82-0.99)**	NA	1.04 (0.90-1.20)	NA
Marcacuzco et al[21] (2018)	0.92 (0.84-1.01)	NA	NA	NA
Balzer et al[22] (2020)	0.93 (0.85-1.02)	0.96 (0.93-0.99)	NA	NA
Scheuermann et al[23] (2020)	0.92 (0.84-1.01)	0.96 (0.92-0.99)	NA	NA
Lenihan et al[24] (2021)	NA	NA	NA	NA
So et al[25] (2021)	**0.90 (0.83-0.97)**	NA	NA	NA
Prezelin-Reydit et al[26] (2021)	NA	**0.96 (0.92-1.00)**	NA	NA

Abbreviations: CI, confidence interval; HR, hazard ratio; NA, not applicable; OR, odds ratio.

© 2022 Ngamvichchukorn T et al. *JAMA Network Open.*

eTable 11. Meta-Regression of Primary Outcomes

Covariate	All-Cause Mortality		
	No. of Studies (Reference)	HR (95% CI)†	*P* Value
**Study Characteristics**			
Risk of bias by NOS (per 1 point)	13 (5, 8, 12, 14, 15, 16, 17, 18, 20, 21, 22, 23, 25)	0.96 (0.76 – 1.20)	0.685
Proportion of PD modality (per %)	13 (5, 8, 12, 14, 15, 16, 17, 18, 20, 21, 22, 23, 25)	1.00 (0.98 – 1.02)	0.811
Study population (adult vs. pediatric/mixed cases)	13 (5, 8, 12, 14, 15, 16, 17, 18, 20, 21, 22, 23, 25)	1.17 (0.55 – 2.48)	0.663
Donor type (deceased vs. mixed cases/unspecified)	13 (5, 8, 12, 14, 15, 16, 17, 18, 20, 21, 22, 23, 25)	0.92 (0.47 – 1.81)	0.790
Publication date (before 2015 vs. 2015 to 2022)	13 (5, 8, 12, 14, 15, 16, 17, 18, 20, 21, 22, 23, 25)	1.30 (0.77 – 2.21)	0.296
Sample size (≤1,000 vs. >1,000)	13 (5, 8, 12, 14, 15, 16, 17, 18, 20, 21, 22, 23, 25)	1.11 (0.61 – 2.02)	0.702
Study setting (single-center vs. multicenter)	13 (5, 8, 12, 14, 15, 16, 17, 18, 20, 21, 22, 23, 25)	**1.58 (1.02 – 2.44)**	**0.040**
Study location (European vs. non-European region/international	13 (5, 8, 12, 14, 15, 16, 17, 18, 20, 21, 22, 23, 25)	**1.58 (1.02 – 2.44)**	**0.040**
**Recipient Characteristics**			
Recipient age (mean, per 1 year)	13 (8, 12, 14, 15, 16, 17, 18, 20, 21, 22, 23, 25)	1.02 (0.98 – 1.07)	0.298
Female (per %)	13 (5, 8, 12, 14, 15, 16, 17, 18, 20, 21, 22, 23, 25)	0.99 (0.95 – 1.03)	0.684
BMI, (mean, per 1 kg/m^2)	9 (8, 15, 16, 17, 18, 21, 22, 23, 25)	1.21 (0.81 – 1.81)	0.296
White race, (per %)	5 (5, 8, 12, 20, 25)	1.00 (0.99 – 1.00)	0.335
Etiology of ESKD by glomerulonephritis (per %)	6 (8, 14, 16, 18, 22, 25)	1.01 (0.88 – 1.16)	0.880
Diabetes (per %)	13 (5, 8, 12, 14, 15, 16, 17, 18, 20, 21, 22, 23, 25)	1.01 (0.99 – 1.02)	0.220
Dialysis vintage (mean, per 1 year)	9 (12, 14, 16, 17, 18, 20, 21, 22, 23)	**0.82 (0.70 – 0.98)**	**0.030**
**Donor and Peritransplant Characteristics**			
Donor age (mean, per 1 year)	9 (8, 12, 15, 16, 17, 18, 21, 22, 23)	0.98 (0.92 – 1.03)	0.352
Living donor type (per %)	9 (8, 12, 14, 15, 16, 17, 18, 22, 23)	1.00 (0.97 – 1.03)	0.979
Cold ischemia time (mean, per 1 hr)	9 (8, 12, 15, 16, 17, 18, 21, 22, 23)	0.98 (0.86 – 1.12	0.712
SPKT (per %)	13 (5, 8, 12, 14, 15, 16, 17, 18, 20, 21, 22, 23, 25)	1.00 (0.99 – 1.02)	0.564

†Effect size for each variable of interest reflecting unit change.

Abbreviations: BMI, body mass index; CI, confidence interval; ESKD, end-stage kidney disease; HR, hazard ratio; NA, not applicable; NOS, Newcastle-Ottawa scale; PD, peritoneal dialysis; SPKT, simultaneous pancreas-kidney transplantation.

© 2022 Ngamvichchukorn T et al. *JAMA Network Open.*

eTable 11. Meta-Regression of Primary Outcomes (Continued)

**Covariate**	**Overall Graft Failure**		
	**No. of Studies (Reference)**	**HR (95% CI)**[†]	***P* Value**
**Study Characteristics**			
Risk of bias by NOS (per 1 point)	10 (5, 6, 8, 9, 12, 14, 16, 22, 23, 26)	1.05 (0.99 – 1.10)	0.062
Proportion of PD modality (per %)	10 (5, 6, 8, 9, 12, 14, 16, 22, 23, 26)	1.00 (0.99 – 1.10)	0.747
Study population (adult vs. pediatric/mixed cases)	10 (5, 6, 8, 9, 12, 14, 16, 22, 23, 26)	1.01 (0.91 – 1.13)	0.802
Donor type (deceased vs. mixed cases/unspecified)	10 (5, 6, 8, 9, 12, 14, 16, 22, 23, 26)	1.02 (0.90 – 1.16)	0.713
Publication date (before 2015 vs. 2015 to 2022)	10 (5, 6, 8, 9, 12, 14, 16, 22, 23, 26)	0.97 (0.84 – 1.11)	0.611
Sample size (≤1,000 vs. >1,000)	10 (5, 6, 8, 9, 12, 14, 16, 22, 23, 26)	0.82 (0.47 – 1.41)	0.415
Study setting (single-center vs. multicenter)	10 (5, 6, 8, 9, 12, 14, 16, 22, 23, 26)	1.08 (0.78 – 1.50)	0.589
Study location (European vs. non-European region/international	10 (5, 6, 8, 9, 12, 14, 16, 22, 23, 26)	1.02 (0.90 – 1.16)	0.694
**Recipient Characteristics**			
Recipient age (mean, per 1 year)	9 (6, 8, 9, 12, 14, 16, 22, 23, 26)	1.00 (0.99 – 1.00)	0.604
Female (per %)	10 (5, 6, 8, 9, 12, 14, 16, 22, 23, 26)	**1.01 (1.00 – 1.02)**	**0.024**
BMI, (mean, per 1 kg/m^2)	4 (8, 16, 22, 23)	0.92 (0.07 – 11.36)	0.896
White race, (per %)	3 (5, 8, 12)	1.00 (0.98 – 1.01)	0.308
Etiology of ESKD by glomerulonephritis (per %)	6 (8, 9, 14, 16, 22, 26)	1.01 (0.97 – 1.04)	0.558
Diabetes (per %)	9 (5, 6, 8, 9, 12, 14, 16, 22, 23)	**1.003 (1.001 – 1.006)**	**0.030**
Dialysis vintage (mean, per 1 year)	8 (6, 9, 12, 14, 16, 22, 23, 26)	1.00 (0.95 – 1.05)	0.928
**Donor and Peritransplant Characteristics**			
Donor age (mean, per 1 year)	8 (6, 8, 9, 12, 16, 22, 23, 26)	1.00 (0.99 – 1.00)	0.650
Living donor type (per %)	9 (6, 8, 9, 12, 14, 16, 22, 23, 26)	1.00 (0.99 – 1.01)	0.707
Cold ischemia time (mean, per 1 hr)	8 (6, 8, 9, 12, 16, 22, 23, 26)	0.99 (0.97 – 1.02)	0.559
SPKT (per %)	10 (5, 6, 8, 9, 12, 14, 16, 22, 23, 26)	1.00 (0.98 – 1.01)	0.685

[†]Effect size for each variable of interest reflecting unit change.

Abbreviations: BMI, body mass index; CI, confidence interval; ESKD, end-stage kidney disease; HR, hazard ratio; NA, not applicable; NOS, Newcastle-Ottawa scale; PD, peritoneal dialysis; SPKT, simultaneous pancreas-kidney transplantation.

© 2022 Ngamvichchukorn T et al. *JAMA Network Open.*

eTable 11. Meta-Regression of Primary Outcomes (Continued)

**Covariate**	**Death-Censored Graft Failure**		
	**No. of Studies (Reference)**	**HR (95% CI)**[†]	***P* Value**
**Study Characteristics**			
Risk of bias by NOS (per 1 point)	5 (5, 12, 15, 18, 20)	**1.23 (1.05 – 1.44)**	**0.026**
Proportion of PD modality (per %)	5 (5, 12, 15, 18, 20)	0.98 (0.95 – 1.01)	0.142
Study population (adult vs. pediatric/mixed cases)	5 (5, 12, 15, 18, 20)	NA	NA
Donor type (deceased vs. mixed cases/unspecified)	5 (5, 12, 15, 18, 20)	1.14 (0.50 – 2.57)	0.650
Publication date (before 2015 vs. 2015 to 2022)	5 (5, 12, 15, 18, 20)	0.67 (0.42 – 1.06)	0.070
Sample size (≤1,000 vs. >1,000)	5 (5, 12, 15, 18, 20)	1.62 (0.50 – 5.24)	0.285
Study setting (single-center vs. multicenter)	5 (5, 12, 15, 18, 20)	1.62 (0.50 – 5.24)	0.285
Study location (European vs. non-European region/international	5 (5, 12, 15, 18, 20)	1.62 (0.50 – 5.24)	0.285
**Recipient Characteristics**			
Recipient age (mean, per 1 year)	4 (12, 15, 18, 20)	1.04 (0.94 – 1.16)	0.220
Female (per %)	5 (5, 12, 15, 18, 20)	0.98 (0.94 – 1.03)	0.341
BMI, (mean, per 1 kg/m^2)	3 (12, 15, 18)	NA	NA
White race, (per %)	3 (5, 12, 20)	1.00 (0.96 – 1.05)	0.519
Etiology of ESKD by glomerulonephritis (per %)	1 (18)	NA	NA
Diabetes (per %)	5 (5, 12, 15, 18, 20)	1.01 (0.99 – 1.03)	0.260
Dialysis vintage (mean, per 1 year)	3 (12, 18, 20)	1.52 (0.23 – 10.13)	0.218
**Donor and Peritransplant Characteristics**			
Donor age (mean, per 1 year)	3 (12, 15, 18)	1.01 (0.47 – 2.20)	0.860
Living donor type (per %)	3 (12, 15, 18)	1.01 (0.87 – 1.16)	0.631
Cold ischemia time (mean, per 1 hr)	3 (12, 15, 18)	0.94 (0.53 – 1.69)	0.423
SPKT (per %)	5 (5, 12, 15, 18, 20)	NA	NA

[†]Effect size for each variable of interest reflecting unit change.

Abbreviations: BMI, body mass index; CI, confidence interval; ESKD, end-stage kidney disease; HR, hazard ratio; NA, not applicable; NOS, Newcastle-Ottawa scale; PD, peritoneal dialysis; SPKT, simultaneous pancreas-kidney transplantation.

© 2022 Ngamvichchukorn T et al. *JAMA Network Open.*

eTable 11. Meta-Regression of Primary Outcomes (Continued)

Covariate	Delayed Graft Function		
	No. of Studies (Reference)	OR (95% CI)[†]	*P* Value
**Study Characteristics**			
Risk of bias by NOS (per 1 point)	6 (2, 4, 5, 7, 13, 15)	1.02 (0.95 – 1.11)	0.449
Proportion of PD modality (per %)	5 (4, 5, 7, 13, 15)	0.99 (0.97 – 1.01)	0.135
Study population (adult vs. pediatric/mixed cases)	6 (2, 4, 5, 7, 13, 15)	1.16 (0.43 – 3.11)	0.706
Donor type (deceased vs. mixed cases/unspecified)	6 (2, 4, 5, 7, 13, 15)	1.05 (0.87 – 1.27)	0.516
Publication date (before 2015 vs. 2015 to 2022)	6 (2, 4, 5, 7, 13, 15)	NA	NA
Sample size (≤1,000 vs. >1,000)	6 (2, 4, 5, 7, 13, 15)	1.06 (0.86 – 1.20)	0.495
Study setting (single-center vs. multicenter)	6 (2, 4, 5, 7, 13, 15)	1.06 (0.86 – 1.30)	0.495
Study location (European vs. non-European region/international	6 (2, 4, 5, 7, 13, 15)	1.06 (0.86 – 1.30)	0.495
**Recipient Characteristics**			
Recipient age (mean, per 1 year)	5 (2, 4, 7, 13, 15)	1.00 (0.97 – 1.03)	0.977
Female (per %)	5 (2, 4, 5, 13, 15)	1.00 (0.99 – 1.02)	0.480
BMI, (mean, per 1 kg/m^2)	3 (7, 13, 15)	1.02 (0.55 – 1.89)	0.743
White race, (per %)	2 (2, 5)	NA	NA
Etiology of ESKD by glomerulonephritis (per %)	1 (13)	NA	NA
Diabetes (per %)	2 (5, 15)	NA	NA
Dialysis vintage (mean, per 1 year)	2 (2, 13)	NA	NA
**Donor and Peritransplant Characteristics**			
Donor age (mean, per 1 year)	3 (7, 13, 15)	1.00 (0.84 – 1.20)	0.798
Living donor type (per %)	5 (2, 4, 7, 13, 15)	1.00 (0.99 – 1.01)	0.743
Cold ischemia time (mean, per 1 hr)	4 (2, 4, 7, 15)	0.96 (0.88 – 1.05)	0.216
SPKT (per %)	6 (2, 4, 5, 7, 13, 15)	NA	NA

[†]Effect size for each variable of interest reflecting unit change.

Abbreviations: BMI, body mass index; CI, confidence interval; ESKD, end-stage kidney disease; NA, not applicable; NOS, Newcastle-Ottawa scale; OR, odds ratio; PD, peritoneal dialysis; SPKT, simultaneous pancreas-kidney transplantation.

eTable 12. Publication Bias

Kidney Transplantation Outcomes	No. of Studies (Reference)	*P* Value for Begg's Test	*P* Value for Egger's Test
**Primary Outcomes**			
All-cause mortality	13 (5, 8, 12, 14, 15, 16, 17, 18, 20, 21, 22, 23, 25)	0.951	0.273
Overall graft failure	10 (5, 6, 8, 9, 12, 14, 16, 22, 23, 26)	0.721	0.947
Death-censored graft failure	5 (5, 12, 15, 18, 20)	0.806	0.609
Delayed graft function	6 (2, 4, 5, 7, 13, 15)	1.000	0.562
**Secondary Outcomes**			
Acute rejection	1 (22)	NA	NA
Graft vessel thrombosis	3 (1, 3, 19)	1.000	0.760
Oliguria (not producing urine in the first 24 hours)	1 (2)	NA	NA
de novo heart failure	1 (24)	NA	NA
NODAT	2 (10, 11)	NA	NA

Abbreviations: CI, confidence interval; NA, not applicable; NODAT, new onset diabetes mellitus after transplantation.

© 2022 Ngamvichchukorn T et al. *JAMA Network Open.*

eTable 13. Quality of Evidence Synthesis and GRADE Evidence Profile of Outcomes

Outcomes (PD vs. HD)	No. of Studies (Ref)	Study Design (Sample Size)	Quality Assessment: Required Domains					Other Issues	Finding and Direction (Magnitude) of Effect	Strength of Evidence
			Study Limitations	Directions	Consistency	Precision	Reporting Bias			
All-cause mortality	13 (5, 8, 12, 14, 15, 16, 17, 18, 20, 21, 22, 23, 25)	Non-RCTs (221,815)	High	Direct	Inconsistency	Precise	Undetected	• Duration-response association could not be determined<br>• Present plausible confounding that would decrease the observed effect[†]	• Thirteen non-RCTs studies with a large sample size illustrated high study limitations and inconsistency of evidence findings based on the sensitivity analyses.<br>• The summary pooled HR was 0.92 (95% CI, 0.84-1.01; *P*=0.085), with moderate degree of heterogeneity (I^2, 68.7%).	Very low (trivial)
Overall graft failure	10 (5, 6, 8, 9, 12, 14, 16, 22, 23, 26)	Non-RCTs (209,287)	High	Direct	Inconsistency	Imprecise	Undetected	• Duration-response association could not be determined<br>• Weak strength of association (magnitude of effect)	• Ten non-RCTs studies with a large sample size showed high study limitations, inconsistency, and imprecise, which subjected to the set of sensitivity analysis.<br>• The summary pooled HR was 0.96 (95% CI, 0.92-0.99; *P*=0.024), with moderate degree of heterogeneity (I^2, 37.2%).	Very low (beneficial with PD)

[†]On the basis of the E-value.

Abbreviations: CI, confidence interval; GRADE, Grading of Recommendations Assessment, Development and Evaluation; HD, hemodialysis; HR, hazard ratio; PD, peritoneal dialysis. OR, odds ratio; RCTs, randomized-controlled trials.

© 2022 Ngamvichchukorn T et al. *JAMA Network Open.*

eTable 13. Quality of Evidence Synthesis and GRADE Evidence Profile of Outcomes (Continued)

Outcomes (PD vs. HD)	No. of Studies (Ref)	Study Design (Sample Size)	Quality Assessment: Required Domains					Other Issues	Finding and Direction (Magnitude) of Effect	Strength of Evidence
			Study Limitations	Directions	Consistency	Precision	Reporting Bias			
Death-censored graft failure	5 (5, 12, 15, 18, 20)	Non-RCTs (96,439)	High	Direct	Consistency	Precise	Undetected	• Duration-response association could not be determined<br>• Present plausible confounding that would decrease the observed effect†	• Five non-RCTs studies revealed high study limitations and consistency based on the sensitivity analyses results.<br>• The summary pooled HR was 0.98 (95% CI, 0.85-1.14; P=0.811), with moderate degree of heterogeneity (I^2, 73.7%).	Very low (trivial)
Delayed graft function	6 (2, 4, 5, 7, 13, 15)	Non-RCTs (47,118)	High	Direct	Consistent	Precise	Undetected	• Duration-response association could not be determined	• Six non-RCTs with high study limitations and low degree of heterogeneity (I^2, 10.4%).<br>• The summary pooled OR was 0.73 (95% CI, 0.70-0.76; P<0.001).<br>• The findings were robust with respect to a set of sensitivity analyses.	Low (beneficial with PD)
Acute rejection	1 (22)	Non-RCTs (2,006)	High	Direct	Unknown	Precise	Suspected	• Duration-response association could not be determined	• A single study with high study limitations by Balzer et al (2020)[22] revealed statistical significance (OR, 0.70; 95% CI, 0.51-0.97; P=0.029). However, the uncertainty in terms of prediction interval could not be estimated.	Insufficient data

†On the basis of the E-value.

© 2022 Ngamvichchukorn T et al. *JAMA Network Open.*

Abbreviations: CI, confidence interval; GRADE, Grading of Recommendations Assessment, Development and Evaluation; HD, hemodialysis; HR, hazard ratio; OR, odds ratio; PD, peritoneal dialysis. OR, odds ratio; RCTs, randomized-controlled trials.

© 2022 Ngamvichchukorn T et al. *JAMA Network Open.*

eTable 13. Quality of Evidence Synthesis and GRADE Evidence Profile of Outcomes (Continued)

Outcomes (PD vs. HD)	No. of Studies (Ref)	Study Design (Sample Size)	Quality Assessment: Required Domains					Other Issues	Finding and Direction (Magnitude) of Effect	Strength of Evidence
			Study Limitations	Directions	Consistency	Precision	Reporting Bias			
Graft vessel thrombosis	3 (1, 3, 19)	Non-RCTs (3,084)	High	Direct	Inconsistency	Imprecise	Suspected	• Duration-response association could not be determined<br>• Present plausible confounding that would decrease the observed effect[†]	• Three non-RCTs with high study limitations and imprecision (95% prediction interval, 1.00 x e^{-5}-1.23 x 10^5).<br>• Publication bias cannot be ruled out due to the small number of studies included.	Very low (trivial)
Oliguria (not producing urine in the first 24 hours)	1 (2)	Non-RCTs (9,291)	High	Direct	Unknown	Precise	Suspected	• Duration-response association could not be determined	• A single study with high study limitations by Bleyer et al (1999)[2] illustrated statistical significance (OR, 0.74; 95% CI, 0.62-0.87; *P*<0.001). However, the uncertainty in terms of prediction interval could not be estimated.	Insufficient data
de novo heart failure	1 (24)	Non-RCTs (27,701)	High	Direct	Unknown	Precise	Suspected	• Duration-response association could not be determined	• A single study with a large sample size by Lenihan et al (2021)[24] illustrated statistical significance (HR, 0.84; 95% CI, 0.78-0.91; *P*<0.001). However, the uncertainty in terms of prediction interval could not be estimated.	Insufficient data

[†]On the basis of the E-value.

Abbreviations: CI, confidence interval; GRADE, Grading of Recommendations Assessment, Development and Evaluation; HD, hemodialysis; HR, hazard ratio; OR, odds ratio; PD, peritoneal dialysis. OR, odds ratio; RCTs, randomized-controlled trials.

© 2022 Ngamvichchukorn T et al. *JAMA Network Open.*

eTable 13. Quality of Evidence Synthesis and GRADE Evidence Profile of Outcomes (Continued)

**Outcomes (PD vs. HD)**	**No. of Studies (Ref)**	**Study Design (Sample Size)**	**Quality Assessment: Required Domains**					**Other Issues**	**Finding and Direction (Magnitude) of Effect**	**Strength of Evidence**
			**Study Limitations**	**Directions**	**Consistency**	**Precision**	**Reporting Bias**			
NODAT	2 (10, 11)	Non-RCTs (2,204)	High	Direct	Inconsistency	Imprecise	Suspected	• Duration-response association could not be determined<br>• Present plausible confounding that would decrease the observed effect†	• Two non-RCTs with high study limitations, high heterogeneity (I^2, 81.8%), and imprecision (effect estimates OR, 1.57; 95% CI, 0.56-4.45; *P*=0.393).<br>• Publication bias cannot be ruled out due to the small number of studies included.	Very low (trivial)

†On the basis of the E-value.

Abbreviations: CI, confidence interval; GRADE, Grading of Recommendations Assessment, Development and Evaluation; HD, hemodialysis; NODAT, new onset diabetes mellitus after transplantation; OR, odds ratio; PD, peritoneal dialysis. OR, odds ratio; RCTs, randomized-controlled trials.

© 2022 Ngamvichchukorn T et al. *JAMA Network Open.*

eFigure 1. PRISMA Flow Diagram of the Literature Search and Selection

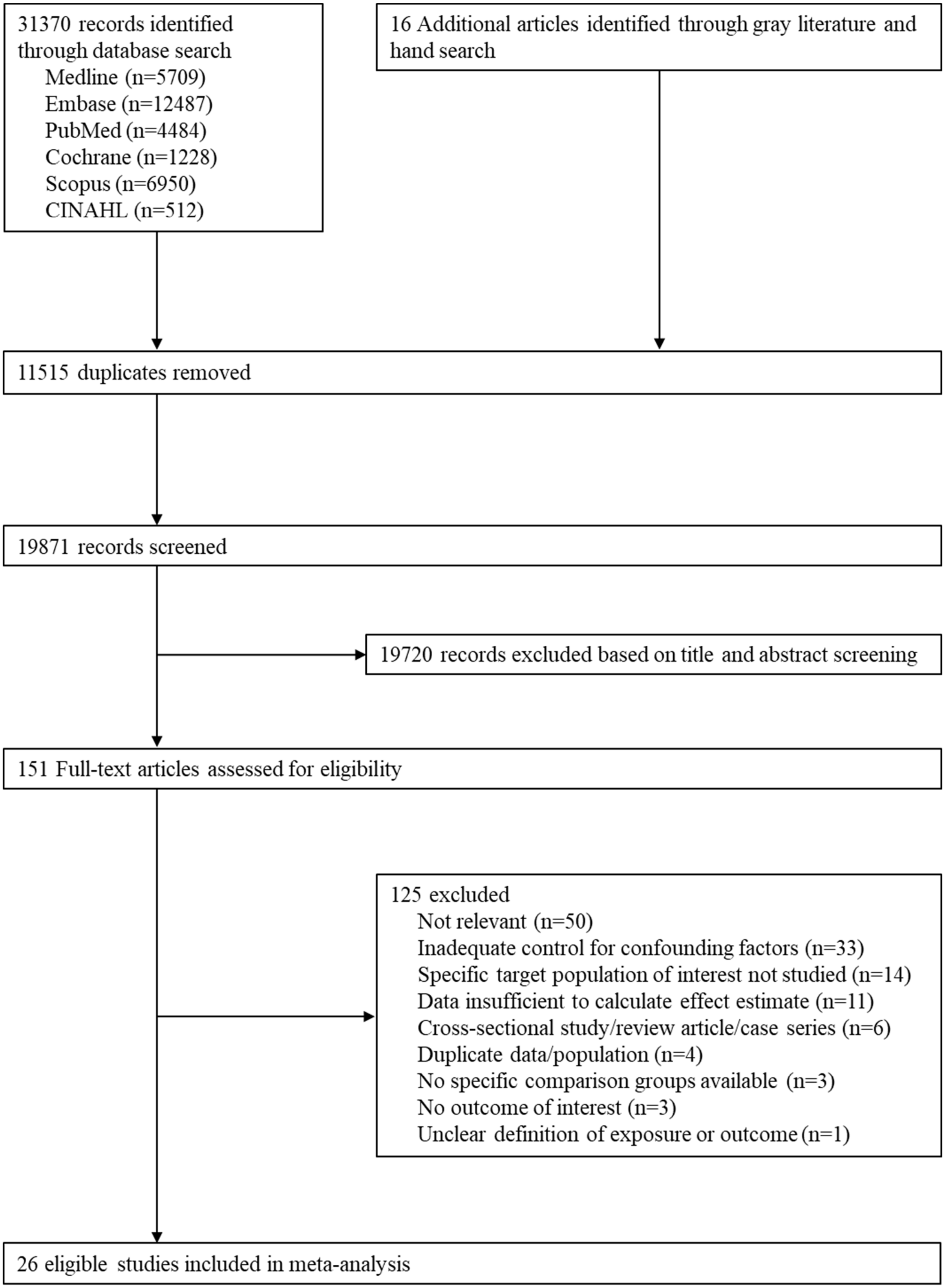

© 2022 Ngamvichchukorn T et al. *JAMA Network Open.*

eFigure 2. Funnel Plot of Included Studies in the Meta-Analysis

A. All-Cause Mortality

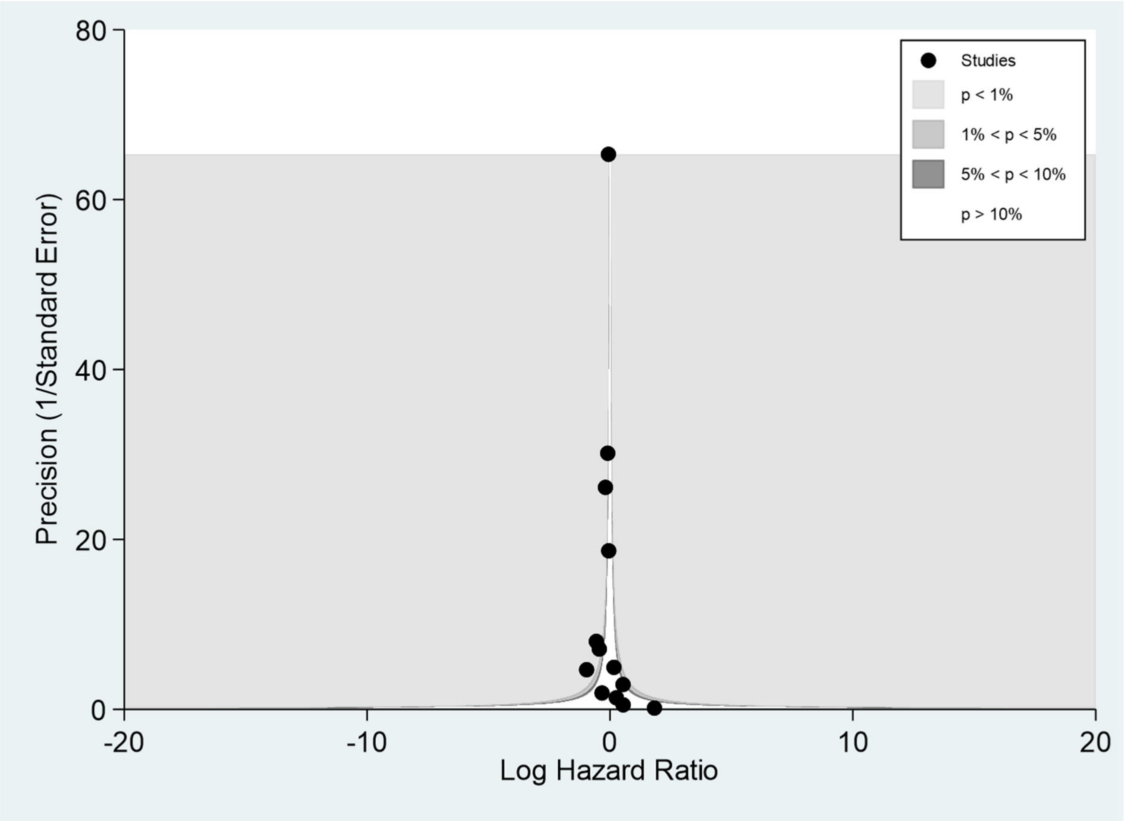

B. Overall Graft Failure

© 2022 Ngamvichchukorn T et al. *JAMA Network Open.*

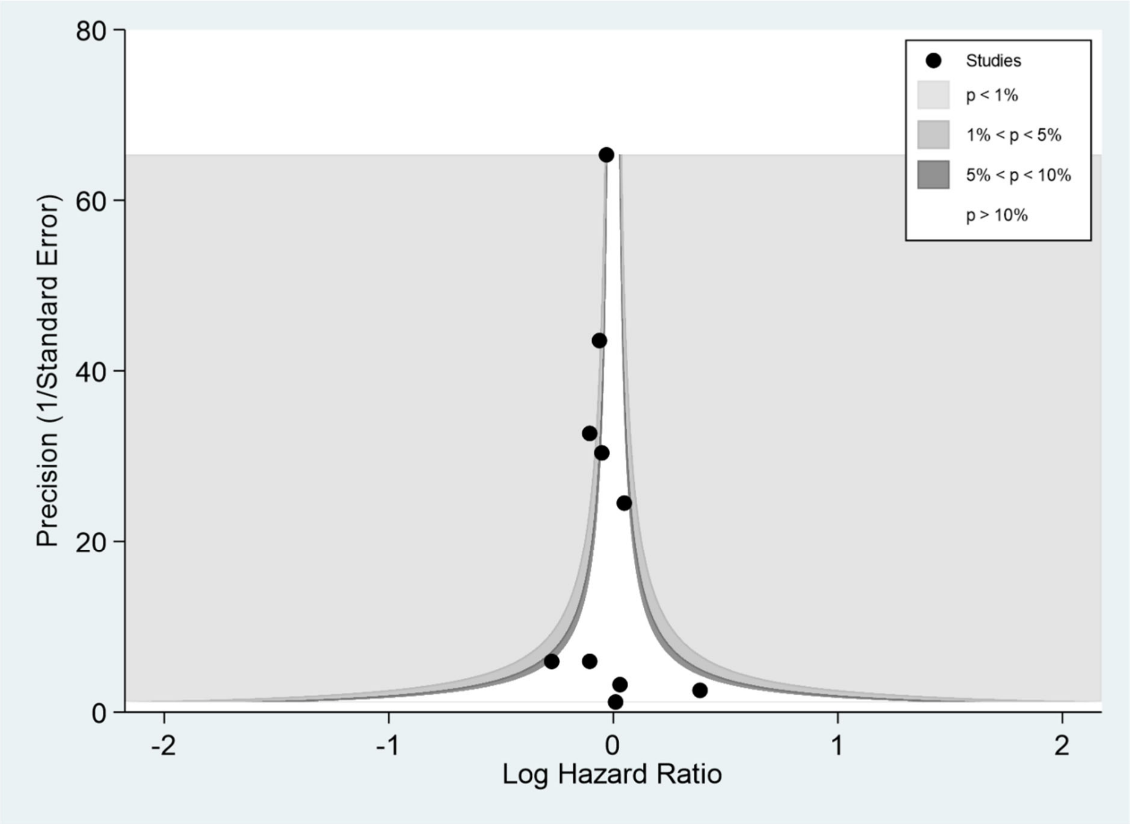

© 2022 Ngamvichchukorn T et al. *JAMA Network Open.*

eFigure 2. Funnel Plot of Included Studies in the Meta-Analysis (Continued)

C. Death-Censored Graft Failure

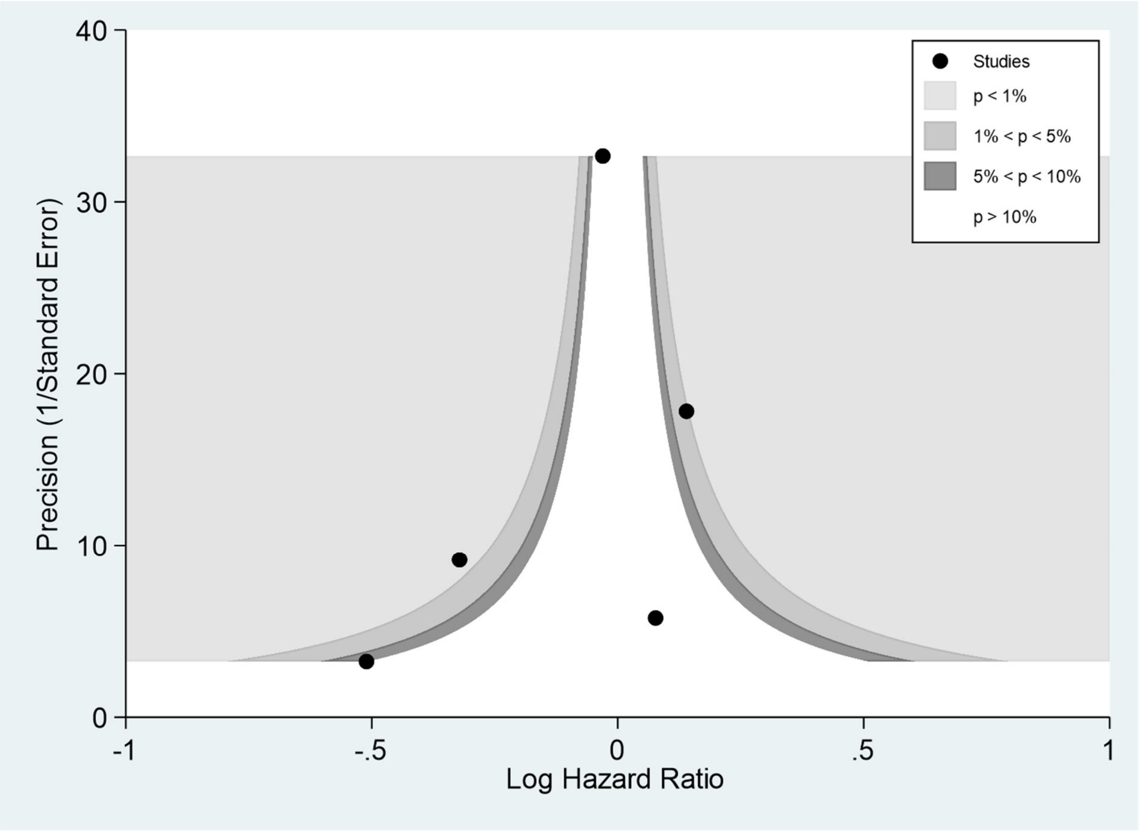

D. Delayed Graft Function

© 2022 Ngamvichchukorn T et al. *JAMA Network Open.*

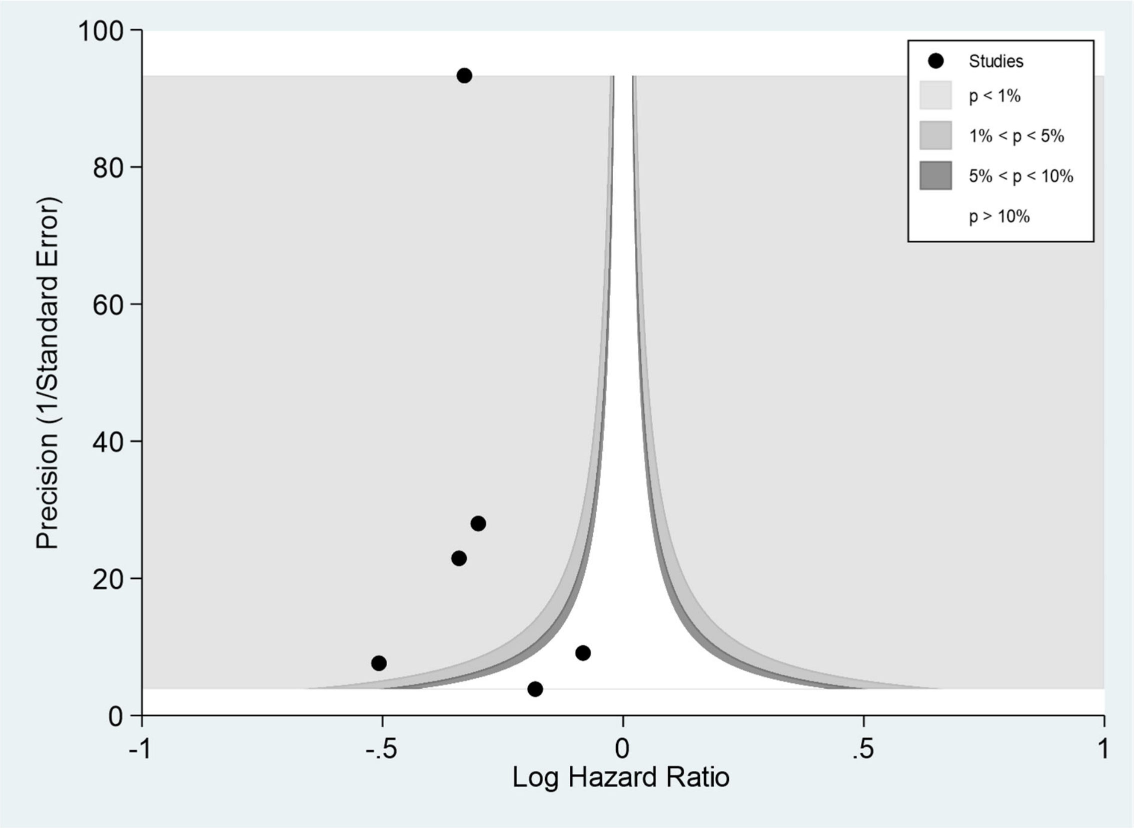

© 2022 Ngamvichchukorn T et al. *JAMA Network Open.*

eReferences

1. Pérez Fontán M, Rodríguez-Carmona A, García Falcón T, Tresancos C, Bouza P, Valdés F: Peritoneal dialysis is not a risk factor for primary vascular graft thrombosis after renal transplantation. Perit Dial Int, 18: 311-316, 1998
2. Bleyer AJ, Burkart JM, Russell GB, Adams PL: Dialysis modality and delayed graft function after cadaveric renal transplantation. J Am Soc Nephrol, 10: 154-159, 1999
3. Ojo AO, Hanson JA, Wolfe RA, Agodoa LY, Leavey SF, Leichtman A, Young EW, Port FK: Dialysis modality and the risk of allograft thrombosis in adult renal transplant recipients. Kidney Int, 55: 1952-1960, 1999
4. Van Biesen W, Vanholder R, Van Loo A, Van Der Vennet M, Lameire N: Peritoneal dialysis favorably influences early graft function after renal transplantation compared to hemodialysis. Transplantation, 69: 508-514, 2000
5. Snyder JJ, Kasiske BL, Gilbertson DT, Collins AJ: A comparison of transplant outcomes in peritoneal and hemodialysis patients. Kidney Int, 62: 1423-1430, 2002
6. Chalem Y, Ryckelynck JP, Tuppin P, Verger C, Chauvé S, Glotz D: Access to, and outcome of, renal transplantation according to treatment modality of end-stage renal disease in France. Kidney Int, 67: 2448-2453, 2005
7. Fontana I, Santori G, Ginevri F, Beatini M, Bertocchi M, Bonifazio L, Saltalamacchia L, Ghinolfi D, Perfumo F, Valente U: Impact of pretransplant dialysis on early graft function in pediatric kidney recipients. Transpl Int, 18: 785-793, 2005
8. Goldfarb-Rumyantzev AS, Hurdle JF, Scandling JD, Baird BC, Cheung AK: The role of pretransplantation renal replacement therapy modality in kidney allograft and recipient survival. Am J Kidney Dis, 46: 537-549, 2005
9. Resende L, Guerra J, Santana A, Mil-Homens C, Abreu F, da Costa AG: Influence of dialysis duration and modality on kidney transplant outcomes. Transplant Proc, 41: 837-839, 2009
10. Courivaud C, Ladrière M, Toupance O, Caillard S, Hurault de Ligny B, Ryckelynck JP, Moulin B, Rieu P, Frimat L, Chalopin JM, Chauvé S, Kazory A, Ducloux D: Impact of pre-transplant dialysis modality on post-transplant diabetes mellitus after kidney transplantation. Clin Transplant, 25: 794-799, 2011
11. Madziarska K, Weyde W, Krajewska M, Patrzalek D, Janczak D, Kusztal M, Augustyniak-Bartosik H, Szyber P, Kozyra C, Klinger M: The increased risk of post-transplant diabetes mellitus in peritoneal dialysis-treated kidney allograft recipients. Nephrol Dial Transplant, 26: 1396-1401, 2011

© 2022 Ngamvichchukorn T et al. *JAMA Network Open.*

12. Schwenger V, Döhler B, Morath C, Zeier M, Opelz G: The role of pretransplant dialysis modality on renal allograft outcome. Nephrol Dial Transplant, 26: 3761-3766, 2011
13. Sezer S, Karakan S, Özdemir Acar FN, Haberal M: Dialysis as a bridge therapy to renal transplantation: comparison of graft outcomes according to mode of dialysis treatment. Transplant Proc, 43: 485-487, 2011
14. Kramer A, Jager KJ, Fogarty DG, Ravani P, Finne P, Pérez-Panadés J, Prütz KG, Arias M, Heaf JG, Wanner C, Stel VS: Association between pre-transplant dialysis modality and patient and graft survival after kidney transplantation. Nephrol Dial Transplant, 27: 4473-4480, 2012
15. Molnar MZ, Mehrotra R, Duong U, Bunnapradist S, Lukowsky LR, Krishnan M, Kovesdy CP, Kalantar-Zadeh K: Dialysis modality and outcomes in kidney transplant recipients. Clin J Am Soc Nephrol, 7: 332-341, 2012
16. López-Oliva MO, Rivas B, Pérez-Fernández E, Ossorio M, Ros S, Chica C, Aguilar A, Bajo MA, Escuin F, Hidalgo L, Selgas R, Jiménez C: Pretransplant peritoneal dialysis relative to hemodialysis improves long-term survival of kidney transplant patients: a single-center observational study. Int Urol Nephrol, 46: 825-832, 2014
17. Martins LS, Malheiro J, Pedroso S, Almeida M, Dias L, Henriques AC, Silva D, Davide J, Cabrita A, Noronha IL, Rodrigues A: Pancreas-Kidney transplantation: Impact of dialysis modality on the outcome. Transpl Int, 28: 972-979, 2015
18. Dipalma T, Fernández-Ruiz M, Praga M, Polanco N, González E, Gutiérrez-Solis E, Gutiérrez E, Andrés A: Pre-transplant dialysis modality does not influence short- or long-term outcome in kidney transplant recipients: analysis of paired kidneys from the same deceased donor. Clin Transplant, 30: 1097-1107, 2016
19. Dębska-Ślizień A, Bobkowska-Macuk A, Bzoma B, Moszkowska G, Milecka A, Zadrożny D, Wołyniec W, Chamienia A, Lichodziejewska-Niemierko M, Król E, Śledziński Z, Rutkowski B: Paired Analysis of Outcomes After Kidney Transplantation in Peritoneal and Hemodialysis Patients. Transplant Proc, 50: 1646-1653, 2018
20. Lin HT, Liu FC, Lin JR, Pang ST, Yu HP: Impact of the pretransplant dialysis modality on kidney transplantation outcomes: a nationwide cohort study. BMJ Open, 8: e020558, 2018
21. Marcacuzco A, Jiménez-Romero C, Manrique A, Calvo J, Cambra F, Caso Ó, García-Sesma Á, Nutu A, Justo I: Outcome of patients with hemodialysis or peritoneal dialysis undergoing simultaneous pancreas-kidney transplantation. Comparative study. Clin Transplant, 32: e13268, 2018

© 2022 Ngamvichchukorn T et al. *JAMA Network Open.*

22. Balzer MS, Pankow S, Claus R, Dumann E, Ruben S, Haller H, Einecke G: Pretransplant dialysis modality and long-term patient and kidney allograft outcome: a 15-year retrospective single-centre cohort study. Transpl Int, 33: 376-390, 2020
23. Scheuermann U, Rademacher S, Jahn N, Sucher E, Seehofer D, Sucher R, Hau HM: Impact of pre-transplant dialysis modality on the outcome and health-related quality of life of patients after simultaneous pancreas-kidney transplantation. Health Qual Life Outcomes, 18: 303, 2020
24. Lenihan CR, Liu S, Airy M, Walther C, Montez-Rath ME, Winkelmayer WC: The Association of Pre-Kidney Transplant Dialysis Modality with de novo Posttransplant Heart Failure. Cardiorenal Med, 11: 209-217, 2021
25. So S, Au EHK, Lim WH, Lee VWS, Wong G: Factors Influencing Long-Term Patient and Allograft Outcomes in Elderly Kidney Transplant Recipients. Kidney Int Rep, 6: 727-736, 2021
26. Prezelin-Reydit M, Madden I, Macher MA, Salomon R, Sellier-Leclerc AL, Roussey G, Lahoche A, Garaix F, Decramer S, Ulinski T, Fila M, Dunand O, Merieau E, Pongas M, Zaloszyc A, Baudouin V, Bérard E, Couchoud C, Leffondré K, Harambat J: Preemptive Kidney Transplantation is Associated With Transplantation Outcomes in Children: Results From the French Kidney Replacement Therapy Registry. Transplantation, 106: 401-411, 2022

© 2022 Ngamvichchukorn T et al. *JAMA Network Open.*
